# Moderate levels of dissolved iron stimulate cellular growth and increase lipid storage in *Symbiodinium* sp.

**DOI:** 10.1111/jpy.70002

**Published:** 2025-03-30

**Authors:** Walter Dellisanti, Swathi Murthy, Elena Bollati, Sara Prehn Sandberg, Michael Kühl

**Affiliations:** ^1^ Marine Biology Section, Department of Biology University of Copenhagen Helsingør Denmark

**Keywords:** cellular physiology, holotomography, iron enrichment, lipid accumulation, Symbiodiniaceae

## Abstract

Dinoflagellates in the family Symbiodiniaceae are fundamental in coral reef ecosystems and facilitate essential processes such as photosynthesis, nutrient cycling, and calcium carbonate production. Iron (Fe) is an essential element for the physiological processes of Symbiodiniaceae, yet its role remains poorly understood in the context of cellular development and metabolic health. Here, we investigated the effect of iron availability—0–100 nM Fe(III)—on *Symbiodinium* sp. ITS2 type A1 cultures and quantified cellular content using flow cytometry and holotomography. Moderate levels of dissolved Fe (50 nM) enhanced growth rates and cellular content development in *Symbiodinium* sp., including lipids and proteins. We observed distinct growth patterns, pigment concentrations, and cellular morphology under increasing Fe concentrations, indicating the influence of iron availability on cellular physiology. Nondestructive, label‐free holotomographic microscopy enabled single‐cell in vivo imaging, revealing higher intracellular lipid accumulation (+57%) in response to 50 nM Fe(III) enrichment. Our findings contribute to a deeper understanding of the relationship between iron availability and *Symbiodinium* sp. growth and cellular development, with potential implications for coral health and reef resilience in the face of environmental stressors.

**Abbreviations**
ANOVA: analysis of variance
AU: absorbance unit
BSA: bovine serum albumin
CDKs: cyclin‐dependent kinases
Chl *a*
: Chlorophyll *a*
DAD: diode array detector
EDTA: ethylenediaminetetraacetic acid
f/2: Guillard's f/2 medium
Fe: iron
Fe(II): ferrous iron
Fe(III): ferric iron
FSC: forward scatter
g: gravitational acceleration
HPLC: high‐performance liquid chromatography
ITS2: Internal Transcribed Spacer 2
K_m_
: Michaelis‐–Menten constant
m: mass
NCBI: National Center for Biotechnology Information
nM: nanomolar
nt: nucleotide
PC5.5: phycoerythrin‐Cy5.5 fluorescence channel
PCR: polymerase chain reaction
PERMANOVA: permutational multivariate analysis of variance
RI: refractive index
ROS: reactive oxygen species
SNP: single nucleotide polymorphism
SSC: side scatter
ULM: universal light meter
US‐SQS/L: universal spherical quantum sensor
V_max_
: maximum growth rate

## INTRODUCTION

Symbiotic dinoflagellates belonging to the family Symbiodiniaceae (LaJeunesse et al., 2018) are of crucial significance to coral reef ecosystems. They play a fundamental role in reef‐building corals via photosynthesis, macro‐ and micronutrient cycling, and calcium carbonate production as the foundation of the reefs (Coffroth & Santos, 2005; Frommlet et al., 2015). However, the current global environmental changes, including ocean warming, nutrient pollution, and deoxygenation, affect the cellular dynamics of the coral–dinoflagellate symbiosis by altering, among other processes, nutrient exchange (Johnson et al., 2021; Morris et al., 2019; Rädecker et al., 2021). Disruption of nutrient exchange can lead to the breakdown of symbiosis–or bleaching–ultimately compromising coral survival (Grottoli et al., 2014).

Among the essential nutrients, iron (Fe) plays a fundamental role in the physiology of Symbiodiniaceae and other microalgae (Reich et al., 2020). Iron is typically present in seawater in nanomolar (nM) concentrations, and it is considered a trace metal (Entsch et al., 1983) that is essential for metabolic processes such as photosynthesis, phagocytosis, and prey digestion in mixotrophic dinoflagellates (Reich et al., 2020; Rodriguez et al., 2016). Although Fe concentration refers to the total amount of Fe present, its bioavailability refers to the fraction of Fe that is accessible and usable by the organism. The balance between Fe concentration and its bioavailability directly influences the homeostasis of photosynthetic organisms, as insufficient Fe availability can limit growth, while excess Fe can induce toxicity (Reich et al., 2020; Romero et al., 2022). Iron is an enzyme cofactor in electron transfer and catalysis (Balk & Schaedler, 2014; Müller, 2023), and its limitation can lead to reduced chlorophyll synthesis, resulting in decreased pigment content and reduced photosynthetic efficiency in Symbiodiniaceae (Iglic, 2011), phytoplankton (Koch & Trimborn, 2024), and freshwater green microalgae (Yadavalli et al., 2012). Iron limitation can also induce the expression of Fe transporters and siderophore production to enhance Fe acquisition from the environment (Sandy & Butler, 2009). Recent observations have suggested that the photochemical performance of Symbiodiniaceae is enhanced in association with *Marinobacter* sp. and *Labrenzia alexandrii* bacteria, which may support nutrient exchange and siderophore production to bind Fe into bioavailable forms (Amin et al., 2009; Matthews, Hoch, et al., 2023; Matthews, Khalil, et al., 2023). In contrast, excessFe can lead to stressful conditions in *Symbiodinium* spp., potentially leading to the production of reactive oxygen species (ROS) through Fenton chemistry and causing oxidative stress (Deleja et al., 2022; Wietheger et al., 2018). This, in turn, can disrupt cellular homeostasis, damage cellular components (such as proteins and lipids), and inhibit the photosynthetic electron transport chain (Rai et al., 2021; Reich et al., 2023). Despite its importance, only a few Symbiodiniaceae species have been extensively studied regarding their Fe requirements (Rodriguez et al., 2016; Reich et al., 2020, 2021).

Iron availability can also affect the lipid contents and lipid profiles of microalgae (Liu et al., 2008; Wang et al., 2023). Lipids are fundamental components of cells and serve in the cellular metabolism of microalgae, including Symbiodiniaceae (Garrett et al., 2013; Kneeland et al., 2013; Pasaribu et al., 2024). They contribute to energy reserves and form integral components of cellular membranes (Patton & Burris, 1983; Tchernov et al., 2004). These functions have implications for nutrient stress responses, energy storage, and proliferation rates of Symbiodiniaceae in nitrogen‐limited host tissue, directly affecting lipid accumulation (Wang et al., 2015). Quantification of lipids and other cellular contents (e.g., protein) in relation to inorganic Fe concentration is therefore relevant in understanding the potential role of this micronutrient in optimizing Symbiodiniaceae cellular conditions and its role in influencing the health and resilience of Symbiodiniaceae populations, both free‐living and *in hospite*.

Recently, optical diffraction tomography (also known as holotomography) techniques have emerged, providing quantitative morphological and biochemical information about individual cells and tissues without the need for exogenous labeling agents (Kim et al., 2024). The use of interferometry allows the measurement of complex optical fields of diffracted light from biological samples, enabling the mapping of the refractive index (RI; Lee et al., 2013; Popescu, 2011). The RI is an optical property that varies with the composition and density of cellular components, enabling the identification and quantification of intracellular structures. Holotomography measurements of phase distortions of light from various incident angles enable the construction of 3‐D refractive index distributions in cells and tissues at submicron resolution (Choi et al., 2007; Habaza et al., 2017), which can then be segmented to identify particular cellular structures with characteristic RI. For example, lipids typically have a higher RIs than other cellular components, and this allows their detection and quantification within live cells (Jung et al., 2018). Holotomography thus provides a rapid, quantitative method for intracellular lipid detection in live cells (Kim et al., 2016; Park et al., 2020). It has been used as an effective imaging technique for studying biological samples including microalgae (Jung et al., 2018), phytoplankton (Lee et al., 2014), bacteria (Bennet et al., 2017; Kim, Zhou, et al., 2014), yeast (Habaza et al., 2015; Rappaz et al., 2009), and red blood cells (Kim, Shim, et al., 2014; Kim, Yoon, et al., 2014). Recent advancements in holotomography have further expanded the potential of this approach by enabling the study of subcellular components, such as lipid droplets, without the need for fixation or staining methods (Kim et al., 2024). This allows researchers to investigate cellular structures and contents in vivo, providing insights into dynamic cellular processes that conventional staining techniques may not cover. However, potential applications of this technique as a non‐invasive, label‐free, in vivo approach to quantify cellular content in Symbiodiniaceae have not yet been explored.

In this study, we investigated the effect of iron enrichment on a *Symbiodinium* sp. strain and quantified its cellular content using a range of label‐free, in vivo techniques, such as flow cytometry and holotomography. The objectives of this study were to (i) evaluate the impact of varying concentrations of dissolved Fe(III) on the growth rate and cellular content development of *Symbiodinium* sp.; (ii) visualize and quantify cellular structures, including proteins and lipids, under different Fe enrichment conditions through holotomography techniques; and (iii) investigate the role of inorganic iron in optimizing cellular growth conditions. We hypothesized that controlled Fe enrichment could enhance *Symbiodinium* sp. growth and cellular content.

## MATERIALS AND METHODS

### *Symbiodinium* cultures

A *Symbiodinium* sp. culture (K‐1618) was acquired from the Norwegian Culture Collection of Algae (NORCCA, Norway) and maintained in TL‐30 medium in a 250 mL polycarbonate flask at 20°C with a photoperiod of 12:12 light:dark (L:D) at 100 μmol photons · m^−2^ · s^−1^ (400–700 nm), as measured with a universal light meter (ULM‐500) equipped with a spherical micro quantum sensor (US‐SQS/L, Heinz Walz, Effeltrich, Germany). Subsequently, subcultures were acclimated to standard f/2 medium for 1 month and maintained under the same conditions prior to the start of experiments. All experimental analyses, except for the flow cytometry assay, were performed during the mid‐exponential phase, as determined via cell counts identifying optimal growth and cellular activity (Lee et al., 2015).

**FIGURE 1:**
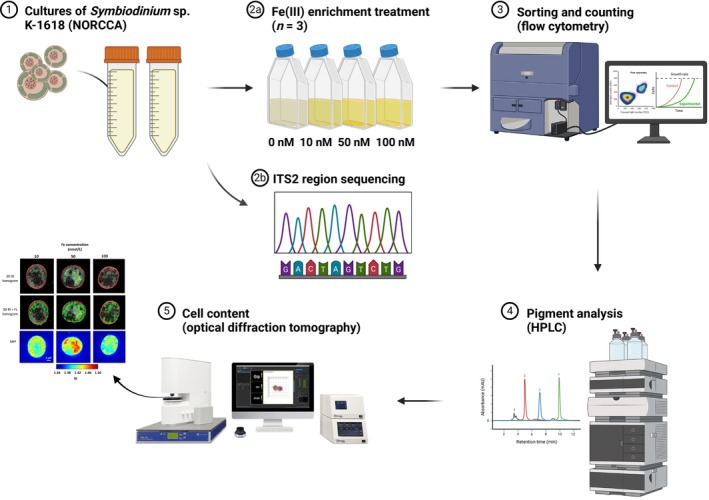
The experimental procedure followed in this study. (1) *Symbiodinium* sp. K‐1618 was acquired from the Norwegian Culture Collection of Algae (NORCCA). (2a) Aliquots of *Symbiodinium* sp. K‐1618 cells were transferred into treatment flasks at increasing levels of inorganic Fe (0, 10, 50, and 100 nM Fe(III)). (2b) The ITS2 rDNA region was sequenced with the Sanger method to genotype the culture. (3) *Symbiodinium* cells were sorted and counted using flow cytometry. (4) Pigment analysis of chlorophyll *a*, carotenoids, and scytonemin was performed with HPLC. (5) Cell content (protein and lipids) was analyzed with optical diffraction tomography in vivo. Created with BioRender.com.

At the start of the experimental period, aliquots of *Symbiodinium* sp. cultures were transferred into each culture flask (20 mL, *n* = 3 replicates per condition) to reach an initial density of ~10,000 cells · mL^−1^, which were maintained under the same conditions as above until they reached the stationary phase (Figure 1). *Symbiodinium* sp. cultures were exposed to increasing iron Fe(III) concentrations (0, 10, 50, and 100 nM total dissolved Fe) at a controlled temperature of 20°C. A dissolved Fe(III) solution (FeCl_3_ · 6H_2_O, Sigma‐Aldrich) was used to enrich the standard f/2 medium to achieve different final Fe concentrations (Reich et al., 2021; Rodriguez et al., 2016). Experimental conditions were prepared as follows: modified f/2 medium with depleted dissolved Fe, 0 nM Fe(III); standard f/2 medium, 10 nM Fe(III); and f/2 media with a concentrated solution of dissolved Fe to reach the initial concentrations of 50 nM Fe(III) and 100 nM Fe(III), respectively.

### Culture identification

To verify the taxonomic assignment of the *Symbiodinium* sp. strain, we performed Sanger sequencing of the ITS2 region of rDNA. Aliquots of the cultures (500,000 cells · mL^−1^) were collected and pelleted by centrifugation during the mid‐exponential growth phase and stored at −80°C. DNA was extracted using the DNAEasy PowerBiofilm kit (Qiagen) following the manufacturer's protocol with slight modifications (all centrifugation steps performed at 8000 × *g* for 90 s). The ITS2 rDNA region was amplified using primers SYM_VAR_5.8S2 and SYM_VAR_REV (Hume et al., 2018) in the following reaction: 0.5 μL of each 10 μM primer, 1 μL of 2 mg · mL^−1^ BSA, 12.5 μL KAPA HiFi HotStart Ready Mix (Roche), 1 μL template DNA (2.65 ng · μL^−1^), and water to a total reaction volume of 25 μL. The amplification cycle was: 98°C for 2 min, followed by 35 cycles of 98°C for 20 s, 61°C for 30 s, 72°C for 30 s, followed by 72°C for 5 min. Successful amplification, amplicon size, and contamination were visually assessed via agarose gel electrophoresis. Polymerase chain reaction (PCR) products were cleaned on AMPure XP beads (Beckman Coulter) and submitted for Sanger sequencing to a commercial provider (Eurofins). Taxonomy was assigned via blastn to the NCBI nucleotide database, as well as local blastn to the SymPortal RefSeq database (github.com/reefgenomics/SymPortal_framework downloaded on March 25, 2024, Hume et al., 2019).

### Growth measurements

*Symbiodinium* cell growth was assessed every third day with a CytoFLEX cytometer (Beckman Coulter, USA), which was equipped with a 50‐mW laser (excitation = 488 nm, fluorescence channel 690/50 nm BP). Flow cytometry was performed throughout the light phase of the L:D cycle until cultures reached the stationary phase. The instrument gain settings were as follows: forward scatter (FSC) = 200, side scatter (SSC) = 83, and phycoerythrin‐Cy5.5 (PC5.5) = 350 (Galotti et al., 2020). Before each analysis, quality control was implemented, as recommended by the CytoFLEX software. All measurements, including culture media blanks, were conducted using an analyzed 1‐mL sample at a slow rate (10 μL · min^−1^) with a threshold of 300 s or 10,000 events. The gating process relied on FSC and SSC patterns, with only cells displaying a positive signal for photosynthetic pigments (PC5.5) being chosen for downstream analysis, with the aim of minimizing potential contamination from non‐algal particles (Figure S1). Cell counts, size, and shape were determined in vivo by FSC and SSC on unstained cells using the CytExpert software v2.5.

The specific growth rate (μ) for each Fe concentration was calculated as μ = ln(*N*_t_) − ln(*N*_0_)/*t*, where *N*_t_ is the cell count at time *t*, *N*_0_ is the initial cell count, and *t* is the time interval.

The Michaelis–Menten equation: *v* = *V*_max_ × *S*/(*K*_m_ + *S*), where *V*_max_ is the maximum growth rate, *K*_m_ is the substrate concentration at half *V*_max_, and *S* is the substrate (Fe) concentration, was then fit to the specific growth rates to estimate *V*_max_ and *K*_m_. The fitting was performed using the nls function in R v4.2.3 (R Core Team, 2016) using the dplyr package v1.1.2 (Wickham et al., 2023).

### Pigment analysis

Quantitative determination of chlorophyll and pigment content was carried out using high‐performance liquid chromatography (HPLC, 1260 Infinity, Agilent Technologies; Frigaard et al., 2004; Kühl et al., 2020). Aliquots of 1 mL for each culture were collected during the mid‐exponential growth phase and transferred to 1.5‐mL tubes and centrifuged at 5000 × *g*, whereafter the pellet was kept at −80°C until analysis. The pigments were extracted by adding 0.4 mL acetone:methanol (7:2 vol:vol) to each tube, which was briefly vortexed, and then kept on ice for a 2‐min extraction time in darkness. Subsequently, samples were sonicated in an ice‐cooled high‐power ultrasonic bath (Misonix 4000; Qsonica LLC., Newtown, CT) in darkness at 80% power for 60 s, consisting of 10 pulses of 2 s ON and 4 s OFF (with an amplitude setting of 100%), and then centrifuged at 12,000 × *g* for 1 min in a mini centrifuge (MiniSpin, Eppendorf AG, Hamburg, Germany). The supernatant was filtered through a 0.2‐μm pore size syringe filter (Sartorius Minisart SRP 4 filter; Sartorius AG, Goettingen, Germany). Then, 100 μL of the extract was then immediately injected into the HPLC. Pigment extracts were separated and analyzed in the HPLC by a diode array detector (HPLC‐DAD and Agilent 1260 Infinity; Agilent Technologies, Santa Clara, CA) fit with an Ascentis C18 column (25 cm × 4.6 mm, Sigma‐Aldrich cat. no. 581325 U), detecting specific absorption wavelengths of compounds. The extracts were run at a constant column temperature of 30°C for 69 min and a flow rate of 1.0 mL · min^−1^ in a changing gradient of solvent A (methanol:acetonitrile:water, 42:33:25, vol/vol/vol) and solvent B (methanol:acetonitrile:ethylacetate, 50:20:30, vol/vol/vol), where the mobile phase changed linearly from 30% solvent B at the time of injection to 100% at 52 min, staying at 100% for 15 min before returning to 30% within 2 min.

The absolute amount of each component was determined from the integrated chromatographic peak area using the formula: *m* = *F* · *A*/(ε · *d*), where *m* is the amount (mg), *F* is flow rate (1 mL min^−1^), *A* is peak area (AU min), *d* is detector light path (1 cm), and ε is the specific absorption coefficient (AU L · g^−1^ · cm^−1^; Frigaard & Seemann, 2025). Elution profiles from the absorbance detector signal at 664 nm (chlorophyll *a*), 460 nm (carotenoids), and 387 nm (scytonemin) were used to calculate pigment ratios from the derived integrated peak areas for each of the identified pigments of interest, using the manufacturer's software (OpenLAB CDS ChemStation Edition; Agilent Technologies). The components analyzed by HPLC had the following retention times and specific absorption coefficients (Dawson et al., 1986; Garcia‐Pichel et al., 1992; Jeffrey, 1963; Lichtenthaler, 1987): Chl *a*, 47.4 min, 79.0 L · g^−1^ · cm^−1^ at 664 nm; carotenoids, 14–59 min, 250 L · g^−1^ · cm^−1^ at 460 nm; and scytonemin, 47.7 min, 113 L · g^−1^ · cm^−1^ at 387 nm.

### Optical diffraction tomography

Quantitative 3D imaging of individual *Symbiodinium* cells was carried out with an optical diffraction tomography (ODT) system (Tomocube HT‐2H; Tomocube Inc., South Korea). The system has an inbuilt ability to perform fluorescence microscopy in parallel with RI mapping. The system is based on Mach–Zehnder interferometry, employing a diode‐pumped green solid‐state laser (532 nm) as a light source. A spatially modulated hologram of the cell was recorded from the diffracted light by a quantitative phase imaging technique (Popescu, 2011). The 3‐D RI distribution of individual cells was then reconstructed from multiple 2‐D images measured at various illumination angles (controlled by a digital micromirror device), using the Fourier diffraction theorem (Choi et al., 2007). The theoretical spatial resolution of the system is 119 nm and 336 nm for lateral and axial directions, respectively (Kim et al., 2016). From the measured 3‐D RI tomograms, different RI regions were visualized with a unique color and rendered as a 3‐D image. Maximum intensity projection images of the RI tomograms were also generated to visualize the 3‐D data set on a 2‐D plane. Data visualization and rendering were carried out using the manufacturer's software (Tomocube Inc., South Korea).

### Wide‐field fluorescence imaging

To determine the presence of chlorophyll in the *Symbiodinium* cells grown at different Fe concentrations, we used the in‐built wide‐field fluorescence imaging capability of the HT‐2H, using the green channel with excitation and emission wavelengths centered ~475 and 520 nm, respectively. For every cell imaged, fluorescence images were captured immediately after the ODT images. At least 30 cells were imaged for each Fe concentration. The fluorescence image was overlaid on the rendered RI tomograms. Although the system is not equipped with a standard Chl *a* filter set, these settings still enabled clear detection of a Chl *a* fluorescence signal due to strong coupling in Symbiodiniaceae with Chl *c* and peridinin (Zigmantas et al., 2002). To confirm the identification of the high RI regions (RI > 1.46) in the 3‐D tomograms as neutral lipids (see below), aliquots of *Symbiodinium* cell cultures were stained with a Nile Red solution (10 μg · mL^−1^) mixed in 30% ethanol solution according to Storms et al. (2014). About 20 μL of the cell sample solution was sandwiched between a pair of coverslips. Cells were then imaged on the Tomocube HT‐2 using the in‐built wide‐field fluorescence system using the red channel, with an excitation wavelength centered ~575 nm and an emission band of 600–800 nm.

### *Symbiodinium* sp. cellular lipid and protein determination

For single‐cell imaging, *Symbiodinium* cell cultures were diluted with culture medium so that the cell density was low enough to avoid having multiple cells within the imaging field. The regions from the RI tomogram with *n* > 1.46 were identified as lipid droplets, as verified by Nile red staining and from the reported average RI for vegetable oils (Firestone, 1999; Ullmann et al., 1985). Then, cell volume and lipid dry mass were calculated for cells grown under different Fe concentrations. The cell volume was calculated from the cell boundary, as determined in the Tomocube software using Otsu thresholding. For further analysis, only mature cells (indicated by a sphericity value >0.8) were chosen, while dividing cells were disregarded. The lipid and the non‐lipid (mostly protein) regions were segmented out based on the RI, that is, assigning RI < 1.46 as protein and RI > 1.46 as lipid. Here, we assumed proteins to be the major non‐lipid component inside the cell (Zhou et al., 2022). The dry mass of these two cell components was then calculated, as per equation 1, using the parameter RI increment (RII), defined as an increment of RI of the solution per unit increment of the solute concentration (Barer & Joseph, 1954).

(1) $$n\left(x,y,z\right)=n_{m}+\sum_{i}\alpha_{i}C_{i}\left(x,y,z\right)$$

where *n*_m_ is the RI of the medium, and *α*_i_ and *C*_i_ are the RII and concentration of protein or lipid, respectively. The typical RII for proteins is 0.19 mL · g^−1^ (Barer & Joseph, 1954), and for lipids, it is 0.135 mL · g^−1^ (Mashaghi et al., 2008). Integrating the concentration over the volume of the component yields its mass.

All data were log‐transformed and checked for normality using the Shapiro–Wilk test and for homogeneity of variance using Levene's test. When data did not meet the assumptions of normality, a Kruskal–Wallis test was used to compare differences in cell counts, size, chlorophyll fluorescence, and morphology. Permutational analysis of variance (PERMANOVA) with 999 permutations was used to compare differences in cell size (FSC) and complexity (SSC) between conditions. A principal component analysis (PCA) was used to compare all cellular morphology data from tomography analysis between conditions. The lipid:protein ratio was calculated by subtracting the log‐transformed protein concentration from the log‐transformed lipid concentration. To indicate the variability of data around the mean, the coefficient of variation (CV%) was used. All statistical analyses were run in R v4.2.3 (R Core Team, 2016) using the dplyr package v1.1.2 (Wickham et al., 2023) and visualized with the ggplot2 package v3.4.4 (Wickham, 2016).

## RESULTS

### ITS2 rDNA region identification

Sanger sequencing of the ITS2 rDNA region returned two sequence variants separated by a single SNP (Table S1), as detected via manual inspection of the chromatogram. Subsequent blastn taxonomic assignment confirmed the identification of the cultures as *Symbiodinium* sp. The most closely related sequence (98.64% identity, query cover 100%, e‐value 7 × 10^−104^) available from the NCBI nucleotide database had been previously detected from a symbiont of the coral *Montastraea faveolata* collected in the Florida Keys, USA (accession: HQ317739; Granados‐Cifuentes & Rodriguez‐Lanetty, 2011), from a free‐living strain collected from Hawaii, USA (accession: AF184948), and from a free‐living strain of unspecified origin (accession: EU449053). The same sequence was labeled as *Symbiodinium* A1dh in the SymPortal RefSeq database (Hume et al., 2019).

### Cellular growth rate and pigment concentration

**FIGURE 2:**
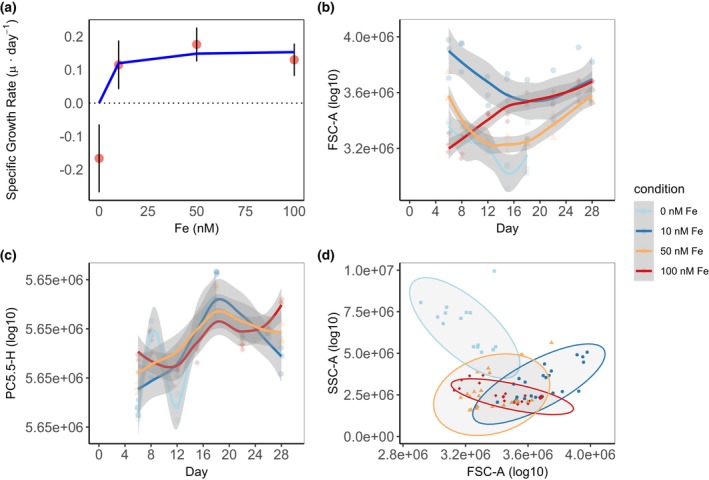
Results of flow cytometric analysis of *Symbiodinium* sp. cells exposed to increasing Fe concentrations (0 to 100 nM Fe). (a) specific growth rate (μ · day^−1^) with predicted curve (in blue) fit to the Michaelis–Menten equation (*V*_max_ = 0.158 · day^−1^, *K*_m_ = 3.19 nM Fe); (b) median values of cell size (FSC‐A); (c) median values of chlorophyll fluorescence (PC5.5‐H); (d) relationship of median cellular size (FSC‐A) versus median complexity (SSC‐A), and ellipses indicating data distribution (95% confidence intervals) in each condition. All values, except for cell abundance, are on a logarithmic scale; the gray area indicates confidence intervals (95%).

The effect of Fe(III) on cell abundance and morphology varied with different Fe concentrations in the culture media. *Symbiodinium* sp. cultures exhibited distinct growth rates when exposed to different Fe concentrations (Kruskal–Wallis test, *χ* ^2^ = 36.76, *df* = 3, *p* < 0.01, Figure 2a; Table S2). Under depleted iron conditions (0 nM), no changes in cell abundance were observed, and fluorescence (PC5.5) was not detected after day 18, indicating the absence of viable cells. Under low (10 nM), moderate (50 nM), and replete (100 nM) Fe(III) concentrations, cultures reached their maximum growth on day 26. The highest abundances were observed in cells growing under 50 nM Fe(III), with a cell density of 1.1 ± 0.04 × 10^6^ cells · mL^−1^. Cells growing under 100 nM iron exhibited the second highest density of 0.81 ± 0.08 × 10^6^ cells · mL^−1^, followed by those growing under 10 nM iron with a concentration of 0.6 ± 0.09 × 10^6^ cells · mL^−1^. The specific growth rates observed were − 0.166 ± 0.1 · day^−1^ under iron‐depleted conditions (0 nM), 0.115 ± 0.05 · day^−1^ at low iron concentration (10 nM), 0.176 ± 0.05 · day^−1^ under moderate iron conditions (50 nM), and 0.130 ± 0.05 · day^−1^ at replete iron levels (100 nM; Figure 2a). The Michaelis–Menten model yielded estimates of *V*_m_ = 0.158 · day^−1^ and *K*_m_ = 3.19 nM, indicating the maximum specific growth rate and the iron concentration required to reach half of *V*_max_, respectively.

Iron concentration also influenced cell size (ANOVA, *F* = 26.24, *p* < 0.01), although no differences were observed between 50 and 100 nM (Figure 2b). Cells growing at 10 nM Fe were, on average, 4.6% and 9.3% larger than those in the 100 and 50 nM treatments, respectively. The depleted Fe condition did not support cellular growth, as the overall cell size at 0 nM Fe was 12.5% smaller than at 10 nM Fe. In the chlorophyll fluorescence signal (Figure 2c), no significant differences were observed among the various Fe concentrations tested. However, in terms of cell size (FSC) and complexity (SSC), significant differences were detected under increasing Fe concentrations (PERMANOVA, *df* = 3, condition; *df* = 83, residual; *F* = 47.773; *p* < 0.01; Figure 2d; Table S3) indicating small and granular cells at 0 nM Fe, large and relatively smooth cells at 10 nM Fe, and medium‐size and smooth cells at 50 and 100 nM Fe, respectively (Figure S2).

Fe(III) concentration affected the concentration of Chl *a*, carotenoids, and scytonemin in *Symbiodinium* sp. cells (Table 1; Table S4). Pigment concentrations of cells grown under depleted Fe concentration (0 nM Fe) were undetectable. Chlorophyll *a* concentration increased from 28.56 ± 3.79 ng · cell^−1^ at 10 nM Fe to 50.57 ± 9.05 ng · cell^−1^ at 100 nM Fe (ANOVA, *df* = 3, condition; *df* = 8, residual; *F* = 47.85; *p* < 0.01). Similarly, the concentration of carotenoids increased from 0.63 ± 0.08 to 1.09 ± 0.19 ng · cell^−1^ in cultures grown in 10 nM Fe and 100 nM Fe, respectively (ANOVA, *df* = 3, condition; *df* = 8, residual; *F* = 50.6; *p* < 0.01). The concentration of scytonemin ranged from 13.88 ± 1.84 ng · cell^−1^ at 10 nM Fe to 24.56 ± 4.39 ng · cell^−1^ at 100 nM Fe (ANOVA, *df* = 3, condition; *df* = 8, residual; *F* = 48.09; *p* < 0.01). The availability of Fe(III) also had a significant impact on cellular volume and content, including proteins and lipids, and on RI (ANOVA, *df* = 3, *F* = 16.34, *p* < 0.01, Figure S3).

**TABLE 1:** Photosynthetic pigments analyzed by HPLC: Chlorophyll *a*, carotenoids, and scytonemin per each condition.

Condition	Fe conc. (nM)	Chlorophyll *a* (Ng · cell^−1^)	Carotenoids (Ng · cell^−1^)	Scytonemin (Ng · cell^−1^)
0 Fe	0	0	0	0
10 Fe	10	28.56 (3.79)	0.63 (0.08)	13.88 (1.84)
50 Fe	50	30.3 (3.48)	0.65 (0.07)	14.72 (1.68)
100 Fe	100	50.57 (9.05)	1.09 (0.19)	24.56 (4.39)

*Note*: All data are expressed as pigment mass per cell of the culture sample. Mean ± standard deviation is shown.

### Mapping RI distributions within *Symbiodinium* cells

**FIGURE 3:**
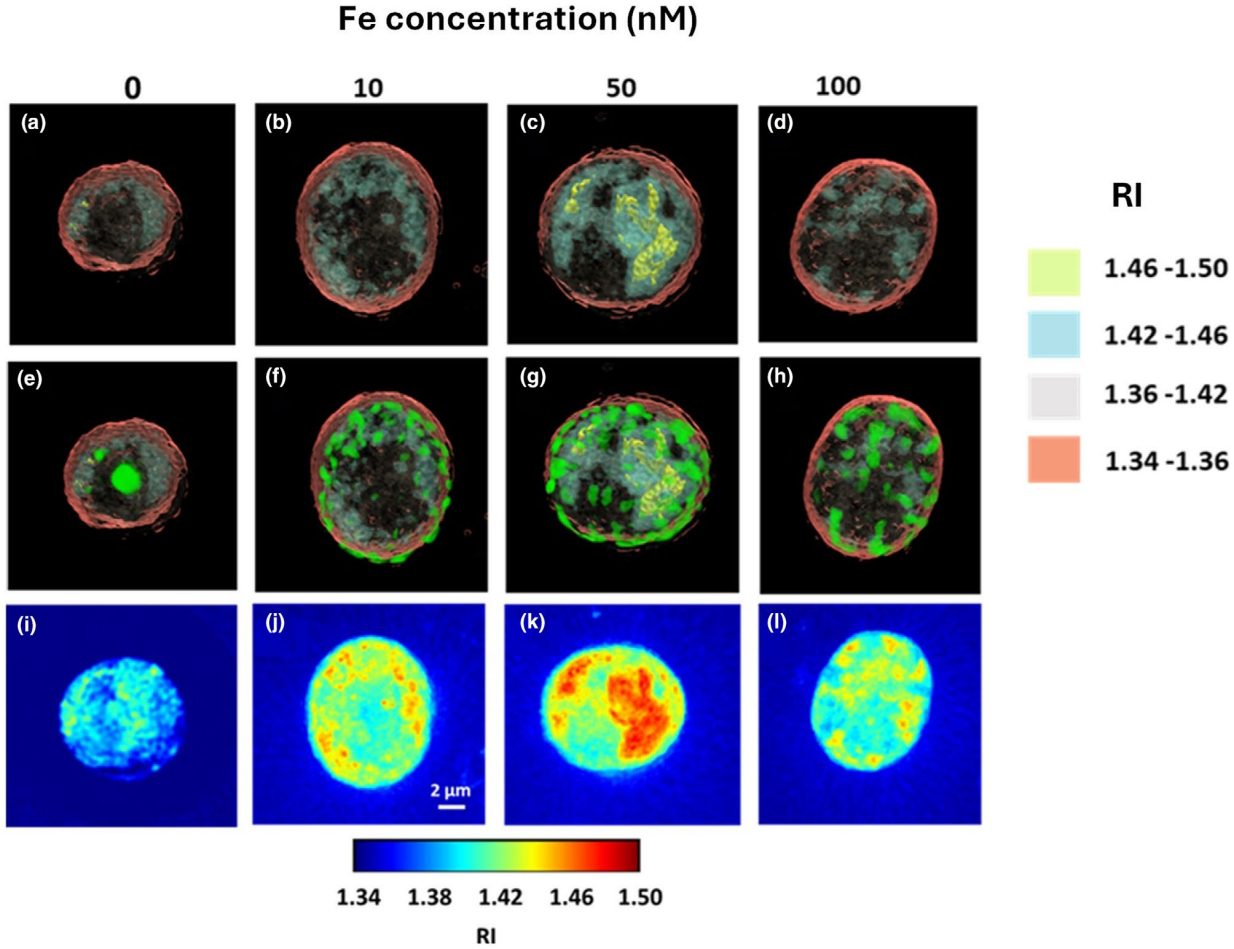
Digital holotomography images of representative *Symbiodinium* cells grown under different Fe concentrations in the culture media. (a–d) 3‐D rendered image of the reconstructed RI distribution. The legend on the right indicates the RI values of the different regions; (e–h) Overlay of chlorophyll fluorescence images on the 3‐D RI rendering; (i–l) Maximum intensity projection images of the tomograms of the reconstructed 3‐D RI distribution.

Optical diffraction tomography (ODT) was employed to map the 3‐D RI distribution of *Symbiodinium* cells. The reconstructed 3‐D RI distribution (Figure 3a–d) and maximum intensity projection images (Figure 3i–l) of cells grown under different Fe (III) concentrations in the culture medium indicated the presence of high RI (>1.46) regions in many of the cells. Based on previous studies (Kim et al., 2016), these regions were suspected to be lipid droplets.

**FIGURE 4:**
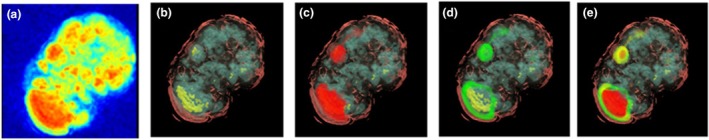
Identification of lipid droplets inside *Symbiodinium* cells: (a) Maximum intensity projection image of the tomogram of the reconstructed 3‐D RI distribution; (b) 3‐D rendered image of the reconstructed RI distribution. The color coding is the same as in the previous image (Figure 3); (c) Overlay of red channel fluorescence image on the 3‐D RI rendering; (d) Overlay of green channel fluorescence image on the 3‐D RI rendering; (e) Overlay of both red and green channel fluorescence images on the 3‐D RI rendering.

To confirm the identification of the high RI (>1.46) regions as lipids, the cells were stained with Nile red dye and imaged using the in‐built wide‐field fluorescence imaging (see methods section). The Nile red fluorescence images (red channel) were compared with the 3‐D RI tomograms of the same cell (Figure 4). The images confirmed that regions with RI >1.46 corresponded to lipid droplets. This is also in accordance with values from the literature for vegetable oils (Ullmann et al., 1985) and for lipid droplets in microalgal cells (Jung et al., 2018). The Nile red and chlorophyll fluorescence signals co‐localized in most cells and were difficult to separate, as Nile red could also stain membranes of organelles like chloroplasts (An et al., 2021; Greenspan et al., 1985). However, in cases of large lipid droplets (as shown in Figure 4, for a dividing cell), it was possible to separate the two signals (to some extent) by appropriate thresholding. The chlorophyll fluorescence (green channel) (Figure 4) appeared as a ring around the lipid droplets. This could, however, be an artifact due to intense light scattering from intracellular components and a possible spillover from Nile red fluorescence.

**FIGURE 5:**
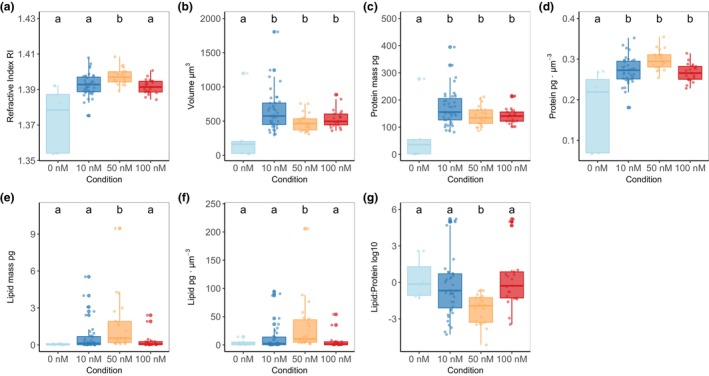
Cellular parameters calculated from digital holotomography imaging for different growth conditions (0–100 nM Fe). (a) mean refractive index (RI); (b) volume of individual *Symbiodinium* sp. cells (μm^3^); (c) cellular protein dry mass (pg); (d) protein concentration per cell volume (pg · μm^−3^); (e) cellular lipid dry mass (pg); (f) lipid concentration per cell volume (pg · μm^−3^); (g) lipid to protein ratio on a log10 scale. Groups with different letters are significantly different (*p* < 0.01, ANOVA with Kruskal‐Wallist test).

To assess the effect of Fe(III) concentration in the culture medium on *Symbiodinium* sp. cell growth, morphology, and biochemical content, the 3‐D RI images were used to quantify (see methods section) cell size, mean RI, protein, and lipid content (Figure 5; Table S5).

Cells grown in 0 nM Fe displayed smaller volume and refractive index (RI), lower lipid concentration and dry mass, as well as reduced protein concentration and dry mass (Figures 3 and 5). In terms of RI, cells grown in 0 nM Fe had a mean value of 1.37 ± 0.02, while the highest mean RI was measured in cells grown in 50 nM Fe condition at 1.4 ± 0.005, compared to 10 and 100 nM conditions (ANOVA, *df* = 3, condition; *df* = 85, residual; *F* = 22.82; *p* < 0.01; Figure 5a, Table S5). The cell volume ranged from 262.9 ± 420.6 μm^3^ in 0 nM Fe to 649.07 ± 298.6 μm^3^ in 10 nM Fe (ANOVA, *df* = 3, condition; *df* = 85, residual; *F* = 23.94; *p* < 0.01; Figure 5b, Table S5), with no significant differences observed in 50 and 100 nM Fe conditions. The lowest protein dry mass was recorded in 0 nM Fe at 61.04 ± 98.55 pg. (ANOVA, *df* = 3, condition; *df* = 85, residual; *F* = 29.05; *p* < 0.01), while the highest was measured in 10 nM Fe at 171.58 ± 66.55 pg, with no significant difference among the other conditions (Figure 5c, Table S5). Protein concentration was lowest in cells grown in 0 nM Fe, at 0.17 ± 0.1 × 10^−4^ pg · μm^−3^ (ANOVA, *df* = 3, condition; *df* = 85, residual; *F* = 22.91; *p* < 0.01), while the highest values were measured in cells grown in 50 nM Fe at 0.3 ± 0.02 × 10^−4^ pg · μm^−3^ (Figure 5d, Table S5). Lipid dry mass and concentration were higher in cells cultured in 50 nM Fe condition than in all other conditions and measured at 1.54 ± 2.2 pg. (Kruskal–Wallis test, *χ* ^2^ = 18.619, *df* = 3, *p* < 0.01) and 31.47 ± 46.61 × 10^−4^ pg · μm^−3^ (Kruskal–Wallis test, *χ* ^2^ = 17.178, *df* = 3, *p* < 0.01), respectively (Figure 5e,f, Table S5). Lipid:protein ratio was higher in cells grown in 50 nM condition at 1.66 ± 0.57 (ANOVA, *df* = 3, condition; *df* = 85, residuals; *F* = 4.82; *p* < 0.01; Figure 5g, Table S5), with no significant difference observed in other conditions.

## DISCUSSION

This study demonstrated that Fe enrichment influences intracellular lipid synthesis in *Symbiodinium* sp. with observable changes in cellular content development. However, this was only true up to an intermediate concentration of 50 nM Fe(III), which seemed to provide the most favorable conditions among the investigated concentrations.

Iron is recognized for its support of the photosynthetic process in coral endosymbionts and its essential role as a cofactor in numerous enzymatic reactions involved in photosynthesis, electron transport, and antioxidant activities (Raven et al., 1999; Reich et al., 2020). Thus, Fe limitation can limit the growth of *Symbiodinium* spp. (Rodriguez and Ho, 2018) as well as of marine phytoplankton (Sunda & Huntsman, 1997), as observed in our experiment for cells grown in depleted iron conditions (0 nM). An increase in the volume of *Symbiodinium* sp. cells was observed at the 10 nM Fe condition, indicating that lower Fe concentrations may facilitate cellular expansion. However, the large variation in cell volume (46%) suggests that cells were at a different growth stage compared to those in other conditions. Iron availability can influence the expression and activity of cell‐cycle regulatory proteins, such as cyclins and cyclin‐dependent kinases (CDKs), which control the progression of cell cycle phases in phytoplankton (Peter & Herskowitz, 1994; Smith et al., 2017). The variability of cell volume in the growth stages might be related to differences in the utilization of Fe within the cells, with varying requirements at different stages of growth. The highest cell growth rate was measured at moderate Fe levels (50 nM Fe), resulting in a smaller cell volume compared to 10 nM (−36%) and 100 nM Fe (−13%). Similar patterns of high growth rate and reduced cell volume were observed in *S. microadriaticum* (ITS2 type A1, Camp et al., 2022), indicating a strict correlation between trace metal availability and cellular growth. However, *in hospite*, symbionts are often nitrogen‐limited (Cui et al., 2022; Falkowski et al., 1993), and enriching iron under coral reef‐like nutrient conditions may exacerbate nitrogen limitation and further unbalance nutrient exchange, photosynthetic activity, and host–symbiont relationship, which could explain the reduced growth rate we observed at high iron levels (100 nM Fe). At the coral host–endosymbiont level, increasing iron levels may stimulate photosynthetic symbiont proliferation, but it could lead to their overgrowth in the coral host tissue, which eventually results in expulsion as a stress response and bleaching. Moreover, the exposure of corals to high iron levels can in turn lead to decreased rates of photosynthesis and reduced maximum quantum yield of PSII (Harland & Brown, 1989; Dellisanti et al., 2024).

Nondestructive, label‐free tomographic imaging represents an advanced tool for investigating the morphology and cellular content of live single cells, such as *Symbiodinium* sp. Using nondestructive holotomography, we measured higher lipid content (+57%), refractive index (+0.35%), and protein concentration (+9%) when *Symbiodinium* sp. was exposed to 50 nM Fe(III), indicating a physiological response to moderate iron concentrations by increasing cellular content. We speculate that moderate levels of Fe(III) enhance the metabolic activity of *Symbiodinium* sp., which leads to increased lipid production to store excess energy as a response mechanism to cellular division. This suggests that there is an optimal Fe(III) concentration in the culture medium, which leads to optimal cellular biochemical content, resulting in the highest growth rates of the *Symbiodinium* cells. Iron plays a critical role in lipid biosynthesis due to its involvement in enzymatic pathways essential for fatty acid production, as observed in marine microalgae (Concas et al., 2021; Polat et al., 2020; Rana & Prajapati, 2021). It is important to note that different *Symbiodinium* species may exhibit distinct strategies for energy storage, reflecting their specific physiological adaptations to their respective ecological niches (Wang et al., 2015). The relatively higher lipid content observed in cells grown at 50 nM Fe(III) may represent a cellular response for energy storage, as observed in *Symbiodinium* cultures and other microalgae (Jiang et al., 2014; Roessler, 1990; Sun et al., 2018). Lipids generate more energy than carbohydrates upon oxidation and can be efficiently packed into the cell, thus providing the best energy reserve for cells to return to homeostatic conditions, particularly in response to stress conditions like temperature fluctuations (Rosset et al., 2019) and nutrient deprivation, such as nitrogen limitation (Jiang et al., 2014).

In coral reef ecosystems, iron availability is closely linked to Symbiodiniaceae response to environmental changes and the resilience of coral–algal symbiosis. Enhanced lipid synthesis in Symbiodiniaceae might increase algal energy reserves, regulating endosymbiosis (Leuzinger et al., 2003; Muscatine et al., 1994) and potentially benefiting coral hosts during thermal stress events (Botana et al., 2022; Imbs & Dembitsky, 2023; Imbs & Yakovleva, 2012). The synthesis of lipids, however, is energetically costly and might result in the relocation of available energy at the expense of other physiological needs such as growth (Botana et al., 2022). Changes in Symbiodiniaceae lipid composition can impact algal physiology, with cascading effects on coral hosts (Matthews et al., 2017). This is particularly relevant in coral bleaching events driven by thermal stress (Hughes et al., 2017) as corals rely on lipid storage for survival under prolonged stress (Liu et al., 2022).

Iron is a limiting nutrient for primary producers, including symbiotic dinoflagellates (Entsch et al., 1983) and eukaryotic marine algae (Greene et al., 1992). However, the concentration of Fe in seawater is affected by mechanisms of scavenging, such as complexation by strong ligands (Johnson et al., 1997). Iron in seawater is present in ferrous Fe(II) and ferric Fe(III) forms, and its speciation depends on oxidation states and thermodynamics (Blain & Tagliabue, 2016). Most of the Fe present in seawater is in the form of the ferric ion Fe(III) and can undergo inorganic speciation to the ferrous ion Fe(II) through hydrolysis and organic speciation with ligands, such as ethylenediaminetetraacetic acid (EDTA) and siderophores produced by marine cyanobacteria (Sandy & Butler, 2009; Blain & Tagliabue, 2016). When considering the bioavailable amount of Fe (Westall et al., 1976), the Fe(III) levels utilized in this study (0–100 nM) were lower compared to previous studies, which used a range of 0–250 nM, corresponding to 0–1250 pM bioavailable Fe (Reich et al., 2020, 2021; Rodriguez & Ho, 2018; Rodriguez et al., 2016). Moreover, in this study, we did not use EDTA as an inorganic ligand to increase the bioavailability of iron, which might account for a lower bioavailable amount of Fe. Despite these methodological differences, previous studies have also highlighted a similar role of Fe(III) in the growth of Symbiodiniaceae, evidenced by higher growth rates when *Symbiodinium microadriaticum* was cultured with Fe(III) complexes (Romero et al., 2022). This response was, however, species‐specific, with other Symbiodiniaceae species responding faster than *S. microadriaticum* (Romero et al., 2022). Iron uptake and intracellular content are also enhanced when *Symbiodinium* spp. are exposed to Fe(III), although high Fe levels reduce the utilization efficiency of this element in *S. microadriaticum*, with a shift to the utilization of other trace metals such as zinc, nickel, and copper to maintain the enzymatic activities and cellular functioning (Blaby‐Haas & Merchant, 2012).

In our study, the higher Chl *a* and carotenoid concentration at 100 nM Fe(III) indicated enhanced pigment production, potentially due to excess Fe stimulating pigment synthesis. Iron is present in almost all the components of the electron transport chain in the chloroplast, including cytochromes, and it is a precursor of chlorophyll synthesis (Pushnik et al., 1984) through hemoproteins in the cytochrome b6f complex (Hogle et al., 2014). The absence of these pigments at 0 nM Fe(III) indicates severe stress or nutritional limitations leading to impaired cellular growth (Rodriguez and Ho, 2018; Romero et al., 2022). However, excessive iron (100 nM Fe(III)) may lead to toxic levels negatively impacting Symbiodiniaceae growth and, in turn, the symbiotic relationships within the coral host (Harland & Brown, 1989; Leigh‐Smith et al., 2018). Moreover, our results indicate that *Symbiodinium* sp. can produce higher levels of scytonemin when cells are grown in 100 nM Fe(III). The biosynthesis of scytonemin in *Symbiodinium* sp. is influenced by osmotic and oxidative stress (Dillon et al., 1999; Liu et al., 2018), potentially serving as an indicator of stress for cells grown under high Fe conditions. This could explain why moderate levels of 50 nM Fe(III) are more favorable, providing sufficient Fe for pigment production, while supporting the development of cellular content without reaching toxic levels (as observed in this study).

In natural coral reef environments, Fe enrichment can exacerbate nitrogen limitation by stimulating Symbiodiniaceae growth, leading to increased nitrogen uptake and further depleting already limited *in hospite* nitrogen resources (Cui et al., 2022; Falkowski et al., 1993; Wang et al., 2015). However, the present study was conducted in an f/2 medium, where nitrogen concentrations far exceeded those in seawater. Thus, the experimental conditions precluded the potential for nitrogen limitation, and the observed decrease in growth under replete Fe levels might not be attributable to nutrient imbalance but rather to physiological limitations such as oxidative stress or disruption in cellular homeostasis. Importantly, it should be noted that the Fe levels utilized in our study (0–100 nM) are higher thanin most natural environments. Coastal waters typically exhibit Fe levels ~ 14.5 nM (Sarthou & Jeandel, 2001), whereas tropical waters often have Fe concentrations below 5 nM (GEOTRACES Intermediate Data Product Group, 2021). Moreover, our study primarily examined the short‐term responses of cultured *Symbiodinium* sp. to increasing Fe concentrations, which may not fully capture the long‐term implications or acclimation potential of these organisms to fluctuating Fe levels in their natural habitats. Additionally, although specific growth rates provide valuable insights, they offer only a partial understanding of overall fitness and ecological performance. Incorporating additional metrics such as specific growth rates, nutrient uptake rates, photophysiological measurements, or gene expression profiles could provide a more comprehensive understanding of the physiological responses of *Symbiodinium* sp. to Fe availability either in culture or *in hospite*.

## CONCLUSIONS

Marine photosynthetic organisms are profoundly influenced by the availability of trace metals, which are fundamental for numerous physiological processes including photosynthesis, cellular homeostasis, and antioxidant enzymatic reactions. Increasing levels of dissolved iron, Fe(III), have been shown to significantly impact the growth of *Symbiodinium* sp., while also stimulating the production of cellular lipids. However, the observed variation in pigment concentrations versus growth and reproduction at different iron concentrations in Symbiodiniaceae cultures suggests that Fe availability and pigment concentration are just one facet of the complex network of factors influencing their physiology. Our study highlights the significance of Fe availability as an essential trace metal in regulating the growth of *Symbiodinium* sp. It emphasizes the necessity of considering the potential role of trace metals when investigating the response of these organisms to environmental changes. Moreover, it highlights the need for further research to elucidate the specific mechanisms and regulatory pathways involved in *Symbiodinium* sp. responses to Fe availability. For future studies, it would be valuable to incorporate measurements, for example, of cellular scavenging of ROS levels under environmental stress, such as ocean warming, and under nitrogen and phosphorus limitation. Additionally, exploring the role of inorganic Fe in supporting thermo‐tolerance may provide insights into the mechanisms of metal availability in the growth of Symbiodiniaceae.

## AUTHOR CONTRIBUTIONS

**Walter Dellisanti:** Conceptualization (lead); data curation (lead); funding acquisition (equal); investigation (equal); methodology (equal); project administration (equal); visualization (equal); writing – original draft (lead); writing – review and editing (equal). **Swathi Murthy:** Conceptualization (lead); formal analysis (lead); investigation (equal); software (equal); visualization (equal); writing – original draft (lead); writing – review and editing (equal). **Elena Bollati:** Formal analysis (equal); investigation (equal); writing – review and editing (equal). **Sara Prehn Sandberg:** Formal analysis (equal); investigation (equal). **Michael Kühl:** Funding acquisition (equal); project administration (equal); supervision (lead); writing – review and editing (equal).

## FUNDING INFORMATION

This research was supported by the European Union (Grant Agreement no. 101062810, MedCorP; WD), the Villum Foundation (Grant no. VIL57413; MK), the Carlsberg Foundation (Grant no. CF21‐0599; MK), and the Gordon and Betty Moore Foundation (Grant no. GBMF9206; https://doi.org/10.37807/GBMF9206; MK). The funders had no role in study design, data collection and analysis, decision to publish, or preparation of the manuscript.

## Supporting information


**Table S1.** Two sequence variances were returned from Sanger sequencing of the ITS2 rDNA region, separated by a single SNP. Bases highlighted in red indicate an SNP detected from a visual inspection of the chromatogram.
**Table S2.** Resume of Kruskal–Wallis test for cell count; analysis of variance (ANOVA) for FSC‐A and PC55. Chi‐squared = test statistic H; *df* = degrees of freedom.
**Table S3.** Analysis of permutational multivariate analysis of variance (PERMANOVA) for cell growth rate data. *df* = degree of freedom, *SS* = sum of squares, *R* ^2^ = proportion of variance, *F*‐value = F‐statistics.
**Table S4.** Analysis of variance (ANOVA) for pigment analysis. *df* = degree of freedom, *SS* = sum of squares, Mean Sq. = mean of squares, *F*‐value = F‐statistics.
**Table S5.** Analysis of variance (ANOVA) and Kruskal–Wallis test for tomography results. Chi‐squared = test statistic H; *df* = degrees of freedom.
**Figure S1.** Flow cytometry workflow. (A) Microalgal cells were gated based on size (FSC‐A); (B) fluorescence signal (PC5.5‐H); (C) size versus complexity (FSC‐A vs SSC‐A), and (D) fluorescence vs size (PC5.5‐H vs FSC‐A). The gating process was carried out on unstained in vivo cells. All values are on a logarithmic scale, except for cell count.
**Figure S2.** Principal component analysis (PCA) of cellular volume and content data from the tomography assay measured in *Symbiodium* sp. cells growing in different conditions.
**Figure S3.** Digital holotomography images of *Symbiodinium* cells grown under 10 nM Fe(III) concentration in the culture media. (a) 3‐D rendered image of the reconstructed RI distribution, at various viewing angles. The color coding is the same as in Figure 3; (b) Overlay of chlorophyll fluorescence image on the 3‐D RI rendering.

## Data Availability

Data and code for data analysis are available in the Zenodo repository at https://zenodo.org/doi/10.5281/zenodo.13845002.

## References

[jpy70002-bib-0001] Amin, S. A. , Green, D. H. , Hart, M. C. , Küpper, F. C. , Sunda, W. G. , & Carrano, C. J. (2009). Photolysis of iron–siderophore chelates promotes bacterial–algal mutualism. Proceedings of the National Academy of Sciences of the United States of America, 106(40), 17071–17076. 10.1073/PNAS.0905512106 19805106 PMC2761308

[jpy70002-bib-0002] An, J. , Miao, X. , Wang, L. , Li, X. , Liu, X. , & Gao, H. (2021). Visualizing the integrity of chloroplast envelope by rhodamine and Nile red staining. Frontiers in Plant Science, 12, 668.414. 10.3389/fpls.2021.668414 PMC8107281 33981327

[jpy70002-bib-0003] Balk, J. , & Schaedler, T. A. (2014). Iron cofactor assembly in plants. Annual Review of Plant Biology, 65, 125–153. 10.1146/ANNUREV-ARPLANT-050213-035759 24498975

[jpy70002-bib-0004] Barer, R. , & Joseph, S. (1954). Refractometry of living cells. Journal of Cell Science, 95, 399–423.

[jpy70002-bib-0005] Bennet, M. , Gur, D. , Yoon, J. , Park, Y. K. , & Faivre, D. (2017). A bacteria‐based remotely tunable photonic device. Advanced Optical Materials, 5(1), 1600617. 10.1002/ADOM.201600617

[jpy70002-bib-0006] Blaby‐Haas, C. E. , & Merchant, S. S. (2012). The ins and outs of algal metal transport. Biochimica et Biophysica Acta (BBA) – Molecular Cell Research, 1823, 1531–1552. 22569643 10.1016/j.bbamcr.2012.04.010 PMC3408858

[jpy70002-bib-1015] Blain, S. , & Tagliabue, A. (2016). Iron speciation in seawater. In S. Blain & A. Tagliabue (Eds.), Iron cycle in oceans (1st ed., pp. 1–21). ISTE Ltd and John Wiley & Sons, Inc.

[jpy70002-bib-0007] Botana, M. T. , Chaves‐Filho, A. B. , Inague, A. , Güth, A. Z. , Saldanha‐Corrêa, F. , Müller, M. N. , Sumida, P. Y. G. , Miyamoto, S. , Kellermann, M. Y. , Valentine, R. C. , & Yoshinaga, M. Y. (2022). Thermal plasticity of coral reef symbionts is linked to major alterations in their lipidome composition. Limnology and Oceanography, 67(7), 1456–1469. 10.1002/LNO.12094

[jpy70002-bib-0008] Camp, E. F. , Nitschke, M. R. , Clases, D. , de Gonzalez Vega, R. , Reich, H. G. , Goyen, S. , & Suggett, D. J. (2022). Micronutrient content drives elementome variability amongst the Symbiodiniaceae. BMC Plant Biology, 22(1), 1–14. 10.1186/S12870-022-03512-0 35395710 PMC8994382

[jpy70002-bib-0009] Choi, W. , Fang‐Yen, C. , Badizadegan, K. , Oh, S. , Lue, N. , Dasari, R. R. , & Feld, M. S. (2007). Tomographic phase microscopy. Nature Methods, 4(9), 717–719. 10.1038/NMETH1078 17694065

[jpy70002-bib-1002] Coffroth, M. A. , & Santos, S. R. (2005). Genetic diversity of symbiotic dinoflagellates in the genus Symbiodinium. Protist, 156, 19–34. 16048130 10.1016/j.protis.2005.02.004

[jpy70002-bib-0010] Concas, A. , Steriti, A. , Pisu, M. , & Cao, G. (2021). Experimental and theoretical investigation of the effects of iron on growth and lipid synthesis of microalgae in view of their use to produce biofuels. Journal of Environmental Chemical Engineering, 9(4), 105349. 10.1016/j.jece.2021.105349

[jpy70002-bib-0011] Cui, G. , Liew, Y. J. , Konciute, M. K. , Zhan, Y. , Hung, S. H. , Thistle, J. , Gastoldi, L. , Schmidt‐Roach, S. , Dekker, J. , & Aranda, M. (2022). Nutritional control regulates symbiont proliferation and life history in coral‐dinoflagellate symbiosis. BMC Biology, 20, 103. 10.1186/s12915-022-01306-2 35549698 PMC9102920

[jpy70002-bib-0012] Dawson, R. , Elliott, D. , Elliott, W. , & Jones, K. (1986). Data for biochemical research (3rd ed.). Oxford University Press.

[jpy70002-bib-0013] Deleja, M. , Paula, J. R. , Repolho, T. , Franzitta, M. , Baptista, M. , Lopes, V. , Simão, S. , Fonseca, V. F. , Duarte, B. , & Rosa, R. (2022). Effects of hypoxia on coral photobiology and oxidative stress. Biology, 11(7), 1068. 10.3390/BIOLOGY11071068 36101446 PMC9312924

[jpy70002-bib-0014] Dellisanti, W. , Zhang, Q. , Ferrier‐Pagès, C. , & Kühl, M. (2024). Contrasting effects of increasing dissolved iron on photosynthesis and O_2_ availability in the gastric cavity of two Mediterranean corals. PeerJ, 12, e17259. 10.7717/peerj.17259 38699194 PMC11064864

[jpy70002-bib-1017] Dillon, J. G. , & Castenholz, R. W. (1999). Scytonemin, a cyanobacterial sheath pigment, protects against UVC radiation: implications for early photosynthetic life. Journal of Phycology, 35(4), 673–681. 10.1046/J.1529-8817.1999.3540673.X

[jpy70002-bib-1008] Entsch, B. , Sim, R. G. , & Hatcher, B. G. (1983). Indications from photosynthetic components that iron is a limiting nutrient in primary producers on coral reefs. Marine Biology, 73(1), 17–30.

[jpy70002-bib-0015] Falkowski, P. G. , Dubinsky, Z. , Muscatine, L. , & McCloskey, L. (1993). Population control in symbiotic corals. BioScience, 43(9), 606–611. 10.2307/1312147

[jpy70002-bib-0016] Firestone, D. (1999). Physical and chemical characteristics of oils, fats, and waxes (2nd ed.). AOCS Press.

[jpy70002-bib-0017] Frigaard, N. U. , Maresca, J. A. , Yunker, C. E. , Jones, A. D. , & Bryant, D. A. (2004). Genetic manipulation of carotenoid biosynthesis in the green sulfur bacterium *Chlorobium tepidum* . Journal of Bacteriology, 186(16), 5210–5220. 10.1128/JB.186.16.5210-5220.2004 15292122 PMC490927

[jpy70002-bib-0018] Frigaard, N. U. , & Seemann, S. E. (2025). Methane and CO_2_ consumption from a synthetic waste gas by microbial communities in enriched seawater. Carbon Capture Science & Technology, 14, 100324. 10.1016/j.ccst.2024.100324

[jpy70002-bib-1003] Frommlet, J. C. , Sousa, M. L. , Alves, A. , Vieira, S. I. , Suggett, D. J. , & Serôdio, J. (2015). Coral symbiotic algae calcify ex hospite in partnership with bacteria. Proceedings of the National Academy of Sciences of the United States of America, 112(19), 6158–6163. 10.1073/PNAS.1420991112 25918367 PMC4434719

[jpy70002-bib-0019] Galotti, A. , Jiménez‐Gómez, F. , & Parra, G. (2020). Flow cytometry assessment of microalgae physiological alterations under CO_2_ injection. Cytometry, Part A, 97(11), 1136–1144. 10.1002/cyto.a.24028 32427422

[jpy70002-bib-0020] Garcia‐Pichel, F. , Sherry, N. D. , & Castenhoz, R. W. (1992). Evidence for an ultraviolet sunscreen role of the extracellular pigment scytonemin in the terrestrial cyanobacterium *Chiorogleopsis* sp. Photochemistry and Photobiology, 56(1), 17–23. 10.1111/j.1751-1097.1992.tb09596.x 1508978

[jpy70002-bib-0021] Garrett, T. A. , Schmeitzel, J. L. , Klein, J. A. , Hwang, J. J. , & Schwarz, J. A. (2013). Comparative lipid profiling of the cnidarian *Aiptasia pallida* and its dinoflagellate symbiont. PLoS ONE, 8(3), 57975. 10.1371/JOURNAL.PONE.0057975 PMC3587569 23483956

[jpy70002-bib-0022] GEOTRACES Intermediate Data Product Group . (2021). The GEOTRACES Intermediate Data Product 2021 version 2 (IDP2021v2). NERC EDS British Oceanographic Data Centre NOC. 10.5285/ff46f034-f47c-05f9-e053-6c86abc0dc7e

[jpy70002-bib-0023] Granados‐Cifuentes, C. , & Rodriguez‐Lanetty, M. (2011). The use of high‐resolution melting analysis for genotyping *Symbiodinium* strains: A sensitive and fast approach. Molecular Ecology Resources, 11(2), 394–399. 10.1111/J.1755-0998.2010.02933.X 21429152

[jpy70002-bib-1013] Greene, R. M. , Geider, R. J. , Kolber, Z. , & Falkowski, P. G. (1992). Iron‐induced changes in light harvesting and photochemical energy conversion processes in eukaryotic marine algae. Plant Physiology, 100, 565–575. 16653030 10.1104/pp.100.2.565 PMC1075596

[jpy70002-bib-0024] Greenspan, P. , Mayer, E. P. , & Fowler, S. D. (1985). Nile red: A selective fluorescent stain for intracellular lipid droplets. Journal of Cell Biology, 100(3), 965–973. 10.1083/jcb.100.3.965 3972906 PMC2113505

[jpy70002-bib-1007] Grottoli, A. G. , Warner, M. E. , Levas, S. J. , Aschaffenburg, M. D. , Schoepf, V. , Mcginley, M. , Baumann, J. , & Matsui, Y. (2014). The cumulative impact of annual coral bleaching can turn some coral species winners into losers. Global Change Biology, 20(12), 3823–3833. 10.1111/GCB.12658 25044878

[jpy70002-bib-0025] Habaza, M. , Gilboa, B. , Roichman, Y. , & Shaked, N. T. (2015). Tomographic phase microscopy with 180 rotation of live cells in suspension by holographic optical tweezers. Optics Letters, 40(8), 1881–1884. 10.1364/OL.40.001881 25872098

[jpy70002-bib-0026] Habaza, M. , Kirschbaum, M. , Guernth‐Marschner, C. , Dardikman, G. , Barnea, I. , Korenstein, R. , Duschl, C. , & Shaked, N. T. (2017). Rapid 3D refractive‐index imaging of live cells in suspension without labeling using dielectrophoretic cell rotation. Advanced Science, 4(2), 1600205. 10.1002/ADVS.201600205 28251046 PMC5323858

[jpy70002-bib-1012] Harland, A. D. , & Brown, B. E. (1989). Metal tolerance in the scleractinian coral *Porites lutea* . Marine Pollution Bulletin, 20, 353–357.

[jpy70002-bib-0028] Hogle, S. L. , Barbeau, K. A. , & Gledhill, M. (2014). Heme in the marine environment: From cells to the iron cycle. Metallomics, 6(6), 1107–1120. 10.1039/C4MT00031E 24811388

[jpy70002-bib-0029] Hughes, T. P. , Barnes, M. L. , Bellwood, D. R. , Cinner, J. E. , Cumming, G. S. , Jackson, J. B. C. , Kleypas, J. , van de Leemput, I. A. , Lough, J. M. , Morrison, T. H. , Palumbi, S. R. , van Nes, E. H. , & Scheffer, M. (2017). Coral reefs in the Anthropocene. Nature, 546(7656), 82–90. 10.1038/nature22901 28569801

[jpy70002-bib-0030] Hume, B. C. C. , Smith, E. G. , Ziegler, M. , Warrington, H. J. M. , Burt, J. A. , LaJeunesse, T. C. , Wiedenmann, J. , & Voolstra, C. R. (2019). SymPortal: A novel analytical framework and platform for coral algal symbiont next‐generation sequencing ITS2 profiling. Molecular Ecology Resources, 19(4), 1063–1080. 10.1111/1755-0998.13004 30740899 PMC6618109

[jpy70002-bib-0031] Hume, B. C. C. , Ziegler, M. , Poulain, J. , Pochon, X. , Romac, S. , Boissin, E. , de Vargas, C. , Planes, S. , Wincker, P. , & Voolstra, C. R. (2018). An improved primer set and amplification protocol with increased specificity and sensitivity targeting the Symbiodinium ITS2 region. PeerJ, 6, e4816. 10.7717/PEERJ.4816 29844969 PMC5970565

[jpy70002-bib-0032] Iglic, K. K. (2011). Trace metal limitation and its role in oxidative stress of coral algal symbionts; implications for thermally induced coral bleaching events. (Publication No. 282) [Doctoral dissertation, University of Western Ontario]. Electronic Thesis and Dissertation Repository. https://ir.lib.uwo.ca/etd/282

[jpy70002-bib-0033] Imbs, A. B. , & Dembitsky, V. M. (2023). Coral lipids. Marine Drugs, 21(10), 539. 10.3390/md21100539 37888474 PMC10608786

[jpy70002-bib-0034] Imbs, A. B. , & Yakovleva, I. M. (2012). Dynamics of lipid and fatty acid composition of shallow‐water corals under thermal stress: An experimental approach. Coral Reefs, 31, 41–53. 10.1007/s00338-011-0817-4

[jpy70002-bib-0035] Jeffrey, S. (1963). Purification and properties of chlorophyll *c* from *Sargassum flavicans* . The Biochemical Journal, 86, 313–331. 10.1042/bj0860313 13964566 PMC1201755

[jpy70002-bib-0036] Jiang, P. L. , Pasaribu, B. , & Chen, C. S. (2014). Nitrogen‐deprivation elevates lipid levels in *Symbiodinium* spp. by lipid droplet accumulation: Morphological and compositional analyses. PLoS ONE, 9(1), e87416. 10.1371/JOURNAL.PONE.0087416 24475285 PMC3903884

[jpy70002-bib-1014] Johnson, K. S. , Gordon, R. M. , & Coale, K. H. (1997). What controls dissolved iron concentrations in the world ocean? Marine Chemistry, 57, 137–161.

[jpy70002-bib-1004] Johnson, M. D. , Swaminathan, S. D. , Nixon, E. N. , Paul, V. J. , & Altieri, A. H. (2021). Differential susceptibility of reef‐building corals to deoxygenation reveals remarkable hypoxia tolerance. Scientific Reports, 11(1), 1–12. 10.1038/s41598-021-01078-9 34848743 PMC8632909

[jpy70002-bib-0037] Jung, J. H. , Hong, S. J. , Kim, H. B. , Kim, G. , Lee, M. , Shin, S. , Lee, S. Y. , Kim, D. J. , Lee, C. G. , & Park, Y. K. (2018). Label‐free non‐invasive quantitative measurement of lipid contents in individual microalgal cells using refractive index tomography. Scientific Reports, 8(1), 6524. 10.1038/s41598-018-24393-0 29695726 PMC5916925

[jpy70002-bib-0038] Kim, H. , Oh, S. , Lee, S. , Lee, K. S. , & Park, Y. (2024). Recent advances in label‐free imaging and quantification techniques for the study of lipid droplets in cells. Current Opinion in Cell Biology, 87, 102342. 10.1016/J.CEB.2024.102342 38428224

[jpy70002-bib-0039] Kim, K. , Lee, S. , Yoon, J. , Heo, J. , Choi, C. , & Park, Y. (2016). Three‐dimensional label‐free imaging and quantification of lipid droplets in live hepatocytes. Scientific Reports, 6(1), 1–8. 10.1038/srep36815 27874018 PMC5118789

[jpy70002-bib-0040] Kim, K. , Yoon, H. , Diez‐Silva, M. , Dao, M. , Dasari, R. R. , & Park, Y. (2014). Special section on advanced biomedical imaging and sensing: High‐resolution three‐dimensional imaging of red blood cells parasitized by *Plasmodium falciparum* and in situ hemozoin crystals using optical diffraction tomography. Journal of Biomedical Optics, 19(1), 1. 10.1117/1.JBO.19.1.011005 PMC4019420 23797986

[jpy70002-bib-0041] Kim, T. , Zhou, R. , Mir, M. , Babacan, S. D. , Carney, P. S. , Goddard, L. L. , & Popescu, G. (2014). White‐light diffraction tomography of unlabelled live cells. Nature Photonics, 8(3), 256–263. 10.1038/nphoton.2013.350

[jpy70002-bib-0042] Kim, Y. , Shim, H. , Kim, K. , Park, H. J. , Jang, S. , & Park, Y. K. (2014). Profiling individual human red blood cells using common‐path diffraction optical tomography. Scientific Reports, 4(1), 1–7. 10.1038/srep06659 PMC4200412 25322756

[jpy70002-bib-0043] Kneeland, J. , Hughen, K. , Cervino, J. , Hauff, B. , & Eglinton, T. (2013). Lipid biomarkers in *Symbiodinium* dinoflagellates: New indicators of thermal stress. Coral Reefs, 32(4), 923–934. 10.1007/S00338-013-1076-3

[jpy70002-bib-0044] Koch, F. , & Trimborn, S. (2024). Discovery of plasma lipids as potential biomarkers distinguishing breast cancer patients from healthy controls. International Journal of Molecular Sciences, 25(21), 469522. 10.3389/FMARS.2019.00514 PMC11546708 39519111

[jpy70002-bib-0045] Kühl, M. , Trampe, E. , Mosshammer, M. , Johnson, M. , Larkum, A. , Frigaard, N. U. , & Koren, K. (2020). Substantial near‐infrared radiation‐driven photosynthesis of chlorophyll *f*‐containing cyanobacteria in a natural habitat. eLife, 9, e50871. 10.7554/eLife.50871 31959282 PMC6974357

[jpy70002-bib-1001] LaJeunesse, T. C. , Parkinson, J. E. , Gabrielson, P. W. , Jeong, H. J. , Reimer, J. D. , Voolstra, C. R. , & Santos, S. R. (2018). Systematic revision of symbiodiniaceae highlights the antiquity and diversity of coral endosymbionts. Current Biology, 28(16), 2570–2580.e6. 10.1016/j.cub.2018.07.008 30100341

[jpy70002-bib-0046] Lee, E. , Jalalizadeh, M. , & Zhang, Q. (2015). Growth kinetic models for microalgae cultivation: A review. Algal Research, 12, 497–512. 10.1016/J.ALGAL.2015.10.004

[jpy70002-bib-0047] Lee, K. R. , Kim, K. , Jung, J. , Heo, J. H. , Cho, S. , Lee, S. , Chang, G. , Jo, Y. J. , Park, H. , & Park, Y. K. (2013). Quantitative phase imaging techniques for the study of cell pathophysiology: From principles to applications. Sensors, 13(4), 4170–4191. 10.3390/S130404170 23539026 PMC3673078

[jpy70002-bib-0048] Lee, S. , Lee, S. , Kim, K. , Mubarok, A. , Park, Y. , Panduwirawan, A. , Park, H. , & Lee, K. (2014). High‐resolution 3‐D refractive index tomography and 2‐D synthetic aperture imaging of live phytoplankton. Journal of the Optical Society of Korea, 18(6), 691–697. 10.3807/JOSK.2014.18.6.691

[jpy70002-bib-1016] Leigh‐Smith, J. , Reichelt‐Brushett, A. , & Rose, A. L. (2018). The characterization of iron (III) in seawater and related toxicity to early life stages of scleractinian corals. Environmental Toxicology and Chemistry, 37(4), 1104–1114. 10.1002/ETC.4043 29149480

[jpy70002-bib-0049] Leuzinger, S. , Anthony, K. R. N. , & Willis, B. L. (2003). Reproductive energy investment in corals: Scaling with module size. Oecologia, 136, 524–531. 12802676 10.1007/s00442-003-1305-5

[jpy70002-bib-0050] Lichtenthaler, H. K. (1987). Chlorophylls and carotenoids: Pigments of photosynthetic biomembranes. Methods in Enzymology, 148(1987), 350–382.

[jpy70002-bib-0051] Liu, C. , Zhang, Y. , Huang, L. , Yu, X. , Luo, Y. , Jiang, L. , Sun, Y. , Liu, S. , & Huang, H. (2022). Differences in fatty acids and lipids of massive and branching reef‐building corals and response to environmental changes. Frontiers in Marine Science, 9, 882663. 10.3389/FMARS.2022.882663

[jpy70002-bib-1018] Liu, H. , Stephens, T. G. , González‐Pech, R. A. , Beltran, V. H. , Lapeyre, B. , Bongaerts, P. , Cooke, I. , Aranda, M. , Bourne, D. G. , Forêt, S. , Miller, D. J. , van Oppen, M. J. H. , Voolstra, C. R. , Ragan, M. A. , & Chan, C. X. (2018). Symbiodinium genomes reveal adaptive evolution of functions related to coral‐dinoflagellate symbiosis. Communications Biology, 1(1), 1–11. 10.1038/s42003-018-0098-3 30271976 PMC6123633

[jpy70002-bib-0052] Liu, Z. Y. , Wang, G. C. , & Zhou, B. C. (2008). Effect of iron on growth and lipid accumulation in *Chlorella vulgaris* . Bioresource Technology, 99(11), 4717–4722. 10.1016/J.BIORTECH.2007.09.073 17993270

[jpy70002-bib-0053] Mashaghi, A. , Swann, M. , Popplewell, J. , Textor, M. , & Reimhult, E. (2008). Optical anisotropy of supported lipid structures probed by waveguide spectroscopy and its application to study of supported lipid bilayer formation kinetics. Analytical Chemistry, 80(13), 5276. 10.1021/AC800988V 18422336

[jpy70002-bib-0054] Matthews, J. L. , Crowder, C. M. , Oakley, C. A. , Lutz, A. , Roessner, U. , Meyer, E. , Grossman, A. R. , Weis, V. M. , & Davy, S. K. (2017). Optimal nutrient exchange and immune responses operate in partner specificity in the cnidarian‐dinoflagellate symbiosis. Proceedings of the National Academy of Sciences of the United States of America, 114(50), 13194–13199. 10.1073/PNAS.1710733114 29158383 PMC5740609

[jpy70002-bib-0055] Matthews, J. L. , Hoch, L. , Raina, J. B. , Pablo, M. , Hughes, D. J. , Camp, E. F. , Seymour, J. R. , Ralph, P. J. , Suggett, D. J. , & Herdean, A. (2023). Symbiodiniaceae photophysiology and stress resilience is enhanced by microbial associations. Scientific Reports, 13(1), 1–13. 10.1038/s41598-023-48020-9 38007500 PMC10676399

[jpy70002-bib-0056] Matthews, J. L. , Khalil, A. , Siboni, N. , Bougoure, J. , Guagliardo, P. , Kuzhiumparambil, U. , DeMaere, M. , le Reun, N. M. , Seymour, J. R. , Suggett, D. J. , & Raina, J. B. (2023). Coral endosymbiont growth is enhanced by metabolic interactions with bacteria. Nature Communications, 14(1), 1–13. 10.1038/s41467-023-42663-y PMC10611727 37891154

[jpy70002-bib-1005] Morris, L. A. , Voolstra, C. R. , Quigley, K. M. , Bourne, D. G. , & Bay, L. K. (2019). Nutrient availability and metabolism affect the stability of coral–Symbiodiniaceae symbioses. Trends in Microbiology, 27(8), 678–689. 10.1016/j.tim.2019.03.004 30987816

[jpy70002-bib-0057] Müller, B. (2023). Iron transport mechanisms and their evolution focusing on chloroplasts. Journal of Plant Physiology, 288, 154059. 10.1016/J.JPLPH.2023.154059 37586271

[jpy70002-bib-0058] Muscatine, L. , Gates, R. D. , & LaFontaine, I. (1994). Do symbiotic dinoflagellates secrete lipid droplets? Limnology and Oceanography, 39, 925–929.

[jpy70002-bib-0059] Park, S. , Ahn, J. W. , Jo, Y. , Kang, H. Y. , Kim, H. J. , Cheon, Y. , Kim, J. W. , Park, Y. , Lee, S. , & Park, K. (2020). Label‐free tomographic imaging of lipid droplets in foam cells for machine‐learning‐assisted therapeutic evaluation of targeted nanodrugs. ACS Nano, 14(2), 1856–1865. 10.1021/ACSNANO.9B07993 31909985

[jpy70002-bib-0060] Pasaribu, B. , Primadona Purba, N. , Dewanti, L. P. , Pasaribu, D. , Muhammad, A. , Khan, A. , Harahap, S. A. , Syamsuddin, M. L. , Ihsan, Y. N. , Siregar, S. H. , Faizal, I. , Herawati, T. , Irfan, M. , Partogi, T. , Simorangkir, H. , & Kurniawan, T. A. (2024). Lipid droplets in endosymbiotic *Symbiodiniaceae* spp. associated with corals. Plants, 13, 949. 10.3390/PLANTS13070949 38611478 PMC11013053

[jpy70002-bib-0061] Patton, J. S. , & Burris, J. E. (1983). Lipid synthesis and extrusion by freshly isolated zooxanthellae (symbiotic algae). Marine Biology, 75(2–3), 131–136. 10.1007/BF00405995

[jpy70002-bib-0062] Peter, M. , & Herskowitz, I. (1994). Joining the complex: Cyclin‐dependent kinase inhibitory proteins and the cell cycle. Cell, 79, 181–184. 7954786 10.1016/0092-8674(94)90186-4

[jpy70002-bib-0063] Polat, E. , Yüksel, E. , & Altinbas, M. (2020). Effect of different iron sources on sustainable microalgae‐based biodiesel production using *Auxenochlorella protothecoides* . Renewable Energy, 162, 1970–1978. 10.1016/j.renene.2020.09.030

[jpy70002-bib-0064] Popescu, G. (2011). Quantitative phase imaging of cells and tissues (1st ed.). McGraw‐Hill. https://www.accessengineeringlibrary.com/content/book/9780071663427

[jpy70002-bib-0065] Pushnik, J. C. , Miller, G. W. , & Manwaring, J. H. (1984). The role of iron in higher plant chlorophyll biosynthesis, maintenance and chloroplast biogenesis. Journal of Plant Nutrition, 7(1–5), 733–758. 10.1080/01904168409363238

[jpy70002-bib-0066] R Core Team . (2016). R: A language and environment for statistical computing [Computer software]. R Foundation for Statistical Computing, Vienna.

[jpy70002-bib-1006] Rädecker, N. , Pogoreutz, C. , Gegner, H. M. , Cárdenas, A. , & Roth, F. (2021). Heat stress destabilizes symbiotic nutrient cycling in corals. Proceeding of the National Academy of Sciences of the United Stated of America, 118(5), e2022653118. 10.1073/pnas.2022653118 PMC7865147 33500354

[jpy70002-bib-0067] Rai, S. , Singh, P. K. , Mankotia, S. , Swain, J. , & Satbhai, S. B. (2021). Iron homeostasis in plants and its crosstalk with copper, zinc, and manganese. Plant Stress, 1, 100008. 10.1016/J.STRESS.2021.100008

[jpy70002-bib-0068] Rana, M. S. , & Prajapati, S. K. (2021). Resolving the dilemma of iron bioavailability to microalgae for commercial sustenance. Algal Research, 59, 102458. 10.1016/j.algal.2021.102458

[jpy70002-bib-0069] Rappaz, B. , Cano, E. , Colomb, T. , Kuhn, J. , Depeursinge, C. D. , Simanis, V. , Magistretti, P. J. , & Marquet, P. P. (2009). Noninvasive characterization of the fission yeast cell cycle by monitoring dry mass with digital holographic microscopy. Journal of Biomedical Optics, 14(3), 034049. 10.1117/1.3147385 19566341

[jpy70002-bib-0070] Raven, J. A. , Evans, M. C. W. , & Korb, R. E. (1999). The role of trace metals in photosynthetic electron transport in O_2_‐evolving organisms. Photosynthesis Research, 60(2–3), 111–150. 10.1023/A:1006282714942

[jpy70002-bib-0071] Reich, H. G. , Camp, E. F. , Roger, L. M. , & Putnam, H. M. (2023). The trace metal economy of the coral holobiont: Supplies, demands and exchanges. Biological Reviews, 98(2), 623–642. 10.1111/brv.12922 36897260

[jpy70002-bib-0072] Reich, H. G. , Rodriguez, I. B. , LaJeunesse, T. C. , & Ho, T. Y. (2020). Endosymbiotic dinoflagellates pump iron: Differences in iron and other trace metal needs among the Symbiodiniaceae. Coral Reefs, 39(4), 915–927. 10.1007/s00338-020-01911-z

[jpy70002-bib-1010] Reich, H. G. , Tu, W. C. , Rodriguez, I. B. , Chou, Y. , Keister, E. F. , Kemp, D. W. , LaJeunesse, T. C. , & Ho, T. Y. (2021). Iron availability modulates the response of endosymbiotic dinoflagellates to heat stress. Journal of Phycology, 57(1), 3–13. 10.1111/JPY.13078 32996595

[jpy70002-bib-0073] Rodriguez, I. B. , & Ho, T. Y. (2018). SARS‐CoV‐2 infection and venous thromboembolism after surgery: An international prospective cohort study. Anaesthesia, 77(1), 310350. 10.3389/FMICB.2018.00142 PMC8652887 34428858

[jpy70002-bib-1009] Rodriguez, I. B. , Lin, S. , Ho, J. , & Ho, T. Y. (2016). Effects of trace metal concentrations on the growth of the coral endosymbiont *Symbiodinium kawagutii* . Frontiers in Microbiology, 7, 82. 10.3389/FMICB.2016.00082 26903964 PMC4744903

[jpy70002-bib-0074] Roessler, P. G. (1990). Environmental control of Glycerolipid metabolism in microalgae: Commercial implications and future research directions. Journal of Phycology, 26(3), 393–399. 10.1111/J.0022-3646.1990.00393.X

[jpy70002-bib-0075] Romero, J. M. D. , Botana, M. T. , Elias, A. d. C. , Nomura, C. S. , Saldanha‐Corrêa, F. , & Espósito, B. P. (2022). Effect of iron speciation on growth and heat resistance of Symbiodiniaceae. Ocean and Coastal Research, 70, e22016. 10.1590/2675-2824070.21103JMDR

[jpy70002-bib-0076] Rosset, S. , Koster, G. , Brandsma, J. , Hunt, A. N. , Postle, A. D. , & D'Angelo, C. (2019). Lipidome analysis of Symbiodiniaceae reveals possible mechanisms of heat stress tolerance in reef coral symbionts. Coral Reefs, 38, 1241–1253. 10.1007/s00338-019-01865-x

[jpy70002-bib-0077] Sandy, M. , & Butler, A. (2009). Microbial iron acquisition: Marine and terrestrial siderophores. Chemical Reviews, 109(10), 4580–4595. 10.1021/CR9002787 19772347 PMC2761978

[jpy70002-bib-1019] Sarthou, G. , & Jeandel, C. (2001). Seasonal variations of iron concentrations in the Ligurian Sea and iron budget in the Western Mediterranean Sea. Marine Chemistry, 74(2–3), 115–129. 10.1016/S0304-4203(00)00119-5

[jpy70002-bib-0078] Smith, S. R. , Gillard, J. T. F. , Kustka, A. B. , McCrow, J. P. , Badger, J. H. , Zheng, H. , New, A. M. , Dupont, C. L. , Obata, T. , Fernie, A. R. , & Allen, A. E. (2017). Correction: Transcriptional orchestration of the global cellular response of a model pennate diatom to diel light cycling under iron limitation. PLoS Genetics, 13(3), e1006688. 10.1371/journal.pgen.1006688 28355217 PMC5371279

[jpy70002-bib-0079] Storms, Z. J. , Cameron, E. , de la Hoz Siegler, H. , & McCaffrey, W. C. (2014). A simple and rapid protocol for measuring neutral lipids in algal cells using fluorescence. Journal of Visualized Experiments, 87, 51441. 10.3791/51441 PMC4217289 24961928

[jpy70002-bib-0080] Sun, X. M. , Ren, L. J. , Zhao, Q. Y. , Ji, X. J. , & Huang, H. (2018). Microalgae for the production of lipid and carotenoids: A review with focus on stress regulation and adaptation. Biotechnology for Biofuels, 11(1), 272. 10.1186/S13068-018-1275-9 30305845 PMC6171298

[jpy70002-bib-0081] Sunda, W. , & Huntsman, S. (1997). Interrelated influence of iron, light and cell size on marine phytoplankton growth. Nature, 390, 389–392. 10.1038/37093

[jpy70002-bib-0082] Tchernov, D. , Gorbunov, M. Y. , de Vargas, C. , Yadav, S. N. , Milligant, A. J. , Häggblom, M. , & Falkowski, P. G. (2004). Membrane lipids of symbiotic algae are diagnostic of sensitivity to thermal bleaching in corals. Proceedings of the National Academy of Sciences of the United States of America, 101(37), 13531–13535. 10.1073/PNAS.0402907101 15340154 PMC518791

[jpy70002-bib-0083] Ullmann, F. , Gerhartz, W. , Yamamoto, S. Y. , Campbell, F. T. , Pfefferkorn, R. , & Rounsaville, J. F. (1985). Ullmann's encyclopedia of industrial chemistry (Vol. 5). Verlag Chemie.

[jpy70002-bib-0084] Wang, H. , Su, Q. , Zhuang, Y. , Wu, C. , Tong, S. , Guan, B. , Zhao, Y. , & Qiao, H. (2023). Effects of iron valence on the growth, photosynthesis, and fatty acid composition of *Phaeodactylum tricornutum* . Journal of Marine Science and Engineering, 11(2), 316. 10.3390/JMSE11020316

[jpy70002-bib-0085] Wang, L. H. , Chen, H. K. , Jhu, C. S. , Cheng, J. O. , Fang, L. S. , & Chen, C. S. (2015). Different strategies of energy storage in cultured and freshly isolated *Symbiodinium* sp. Journal of Phycology, 51(6), 1127–1136. 10.1111/JPY.12349 26987007

[jpy70002-bib-1020] Westall, J. C. , Zachary, J. L. , & Morel, F. M. M. (1976). MINEQL: A computer program for the calculation of chemical equilibrium composition of aqueous systems . (Technical Note No. 18). https://hdl.handle.net/1721.1/142980

[jpy70002-bib-0087] Wickham, H. (2016). ggplot2: Elegant graphics for data analysis. Springer‐Verlag. https://ggplot2.tidyverse.org

[jpy70002-bib-0088] Wickham, H. , François, R. , Henry, L. , Müller, K. , & Vaughan, D. (2023). dplyr: A grammar of data manipulation. R package version 1.1.4, https://github.com/tidyverse/dplyr; https://dplyr.tidyverse.org

[jpy70002-bib-0089] Wietheger, A. , Starzak, D. E. , Gould, K. S. , & Davy, S. K. (2018). Differential ROS generation in response to stress in *Symbiodinium* spp. The Biological Bulletin, 234(1), 11–21. 10.1086/696977 29694799

[jpy70002-bib-0090] Yadavalli, V. , Jolley, C. C. , Malleda, C. , Thangaraj, B. , Fromme, P. , & Subramanyam, R. (2012). Alteration of proteins and pigments influence the function of photosystem I under iron deficiency from *Chlamydomonas reinhardtii* . PLoS ONE, 7(4), e35084. 10.1371/JOURNAL.PONE.0035084 22514709 PMC3325961

[jpy70002-bib-0091] Zhou, Y. , Liu, L. , Li, M. , & Hu, C. (2022). Algal biomass valorisation to high‐value chemicals and bioproducts: Recent advances, opportunities and challenges. Bioresource Technology, 344, 126371. 10.1016/J.BIORTECH.2021.126371 34838628

[jpy70002-bib-0092] Zigmantas, D. , Hiller, R. G. , Sundström, V. , & Polívka, T. (2002). Carotenoid to chlorophyll energy transfer in the peridinin‐chlorophyll‐a‐protein complex involves an intramolecular charge transfer state. Proceedings of the National Academy of Sciences of the United States of America, 99(26), 16760–16765. 10.1073/PNAS.262537599 12486228 PMC139217

