# Supplementary material for: Moderate levels of dissolved iron stimulate cellular growth and increase lipid storage in Symbiodinium sp
Source: J Phycol. 2025 Mar 30;61(3):558–73. doi: 10.1111/jpy.70002 (PMC12168108; doi:10.1111/jpy.70002)
Supplement: Supplementary file 1 — Table S1. Two sequence variances were returned from Sanger sequencing of the ITS2 rDNA region, separated by a single SNP. Bases highlighted in red indicate an SNP detected from a visual inspection of the chromatogram. Table S2. Resume of Kruskal–Wallis test for cell count; analysis of variance (ANOVA) for FSC‐A and PC55. Chi‐squared = test statistic H; df = degrees of freedom. Table S3. Analysis of permutational multivariate analysis of variance (PERMANOVA) for cell growth rate data. df = degree of freedom, SS = sum of squares, R 2 = proportion of variance, F‐value = F‐statistics. Table S4. Analysis of variance (ANOVA) for pigment analysis. df = degree of freedom, SS = sum of squares, Mean Sq. = mean of squares, F‐value = F‐statistics. Table S5. Analysis of variance (ANOVA) and Kruskal–Wallis test for tomography results. Chi‐squared = test statistic H; df = degrees of freedom. Figure S1. Flow cytometry workflow. (A) Microalgal cells were gated based on size (FSC‐A); (B) fluorescence signal (PC5.5‐H); (C) size versus complexity (FSC‐A vs SSC‐A), and (D) fluorescence vs size (PC5.5‐H vs FSC‐A). The gating process was carried out on unstained in vivo cells. All values are on a logarithmic scale, except for cell count. Figure S2. Principal component analysis (PCA) of cellular volume and content data from the tomography assay measured in Symbiodium sp. cells growing in different conditions. Figure S3. Digital holotomography images of Symbiodinium cells grown under 10 nM Fe(III) concentration in the culture media. (a) 3‐D rendered image of the reconstructed RI distribution, at various viewing angles. The color coding is the same as in Figure 3; (b) Overlay of chlorophyll fluorescence image on the 3‐D RI rendering. [file JPY-61-558-s001.docx]

**Supplementary information**

**Table S1.** Two sequence variances were returned from Sanger sequencing of the ITS2 rDNA, separated by a single SNP. Bases highlighted in red indicate an SNP detected from a visual inspection of the chromatogram.

>Seq1

CTCTTGGGAAATGCCTGAGAGCATGTCTGCTTCAGTGCTTCTACTTTTCCTATTCCTGCTGCTCCTTTCAAGGGGTGGTGCTGCTGTGTGCTACTGCATACTTTGCATTGGCATGCTCAGTATTAAGCGTTGCCCACTGGGTTGACTGATCAACGTTTCATGTCTTTTTCAGTCGGGCAACTCAACACCTGGTGTCTTGAACACTTCCTAGCATGAAGTC

>Seq2

CTCTTGGGATATGCCTGAGAGCATGTCTGCTTCAGTGCTTCTACTTTTCCTATTCCTGCTGCTCCTTTCAAGGGGTGGTGCTGCTGTGTGCTACTGCATACTTTGCATTGGCATGCTCAGTATTAAGCGTTGCCCACTGGGTTGACTGATCAACGTTTCATGTCTTTTTCAGTCGGGCAACTCAACACCTGGTGTCTTGAACACTTCCTAGCATGAAGTC

**Table S2.** Resume of Kruskall-Wallis test for cell count; analysis of variance (ANOVA) for FSC-A and PC55. Chi-squared = test statistic H; *df* = degrees of freedom.

| **Kruskal-Wallis test** | |  |  |  |  |  |  | | |
| --- | --- | --- | --- | --- | --- | --- | --- | --- | --- |
| **Species** | **Parameter** | **N** | **Chi-squared** | ***df*** | ***p*-value** | | |  |  |
| *Symbiodinium sp.* | Count | 9 | 36.76 | 3 | < 0.01 |  |  | | |
| **ANOVA test** |  |  |  |  |  |  |  | | |
| **Species** | **Parameters** |  | ***df*** | ***SS*** | **Mean Sq.** | ***F*-value** | ***p*-value** | | |
| *Symbiodinium sp.* | FSCA | Condition | 3 | 2.27^12^ | 7.58^11^ | 26.24 | < 0.01 | | |
|  |  | Residuals | 83 | 2.4^12^ | 2.89^10^ |  |  | | |
|  | PC55 | Condition | 3 | 1.55^6^ | 5.17^5^ | 1.023 | *n.s.* | | |
|  |  | Residuals | 83 | 4.19^7^ | 5.05^5^ |  |  | | |

**Table S3.** Analysis of permutational multivariate analysis of variance (PERMANOVA) for cell growth rate data. *df* = degree of freedom, *SS* = sum of squares, *R*^2^ = proportion of variance, *F*-value = F-statistics.

|  | ***df*** | ***SS*** | ***R*^2^** | ***F*-value** | ***p*-value** |
| --- | --- | --- | --- | --- | --- |
| Condition | 3 | 0.87 | 0.63 | 47.77 | < 0.01 |
| Residual | 83 | 0.5 | 0.37 |  |  |
| Total | 86 | 1.38 | 1 |  |  |

**Table S4.** Analysis of variance (ANOVA) for pigment analysis. *df* = degree of freedom, *SS* = sum of squares, Mean Sq. = mean of squares, *F*-value = F-statistics.

| **Parameter** |  | ***df*** | ***SS*** | **Mean Sq.** | ***F*-value** | ***p*-value** |
| --- | --- | --- | --- | --- | --- | --- |
| Chl *a* | Condition | 3 | 3.89 | 1.29 | 47.85 | < 0.01 |
|  | Residuals | 8 | 0.22 | 0.02 |  |  |
| Carotenoids | Condition | 3 | 0.002 | 0.001 | 50.6 | < 0.01 |
|  | Residuals | 8 | 0.001 | 0.001 |  |  |
| Scytonemin | Condition | 3 | 0.009 | 0.003 | 48.09 | < 0.01 |
|  | Residuals | 8 | 0.001 | 0.001 |  |  |

**Table S5.** Analysis of variance (ANOVA) and Kruskall-Wallis test for tomography results. Chi-squared = test statistic H; *df* = degrees of freedom.

| **Parameter** |  | ***df*** | ***SS*** | **Mean Sq.** | ***F*-value** | ***p*-value** |
| --- | --- | --- | --- | --- | --- | --- |
| RI | Condition | 3 | 0.0003 | 1.11^-4^ | 22.82 | < 0.01 |
|  | Residuals | 85 | 0.0004 | 4.86^-4^ |  |  |
| Volume | Condition | 3 | 3.55 | 1.183 | 23.94 | < 0.01 |
|  | Residuals | 85 | 4.2 | 0.049 |  |  |
| Protein mass | Condition | 3 | 6.68 | 2.226 | 29.05 | < 0.01 |
|  | Residuals | 85 | 6.51 | 0.076 |  |  |
| Protein conc. | Condition | 3 | 0.56 | 0.188 | 22.91 | < 0.01 |
|  | Residuals | 85 | 0.69 | 0.008 |  |  |
| Lipid:Protein | Condition | 3 | 23.87 | 7.955 | 4.82 | < 0.01 |
|  | Residuals | 85 | 140.16 | 1.649 |  |  |
| *Kruskall-Wallis test* | |  |  |  |  |  |
| **Parameter** | **χ^2^** | ***df*** | ***p*-value** |  |  |  |
| Lipid mass | 18.619 | 3 | < 0.01 |  |  |  |
| Lipid conc. | 17.178 | 3 | < 0.01 |  |  |  |

**
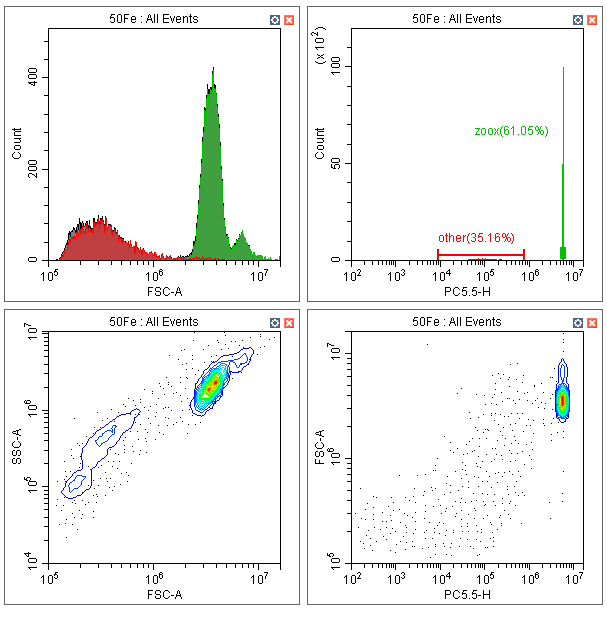
**

**Figure S1**. Flow cytometry workflow. A) microalgal cells were gated based on size (FSC-A); B) fluorescence signal (PC5.5-H); C) size vs complexity (FSC-A vs SSC-A), and D) fluorescence vs size (PC5.5-H vs FSC-A). The gating process was carried out on unstained in-vivo cells. All values are on a logarithmic scale, except for cell count.


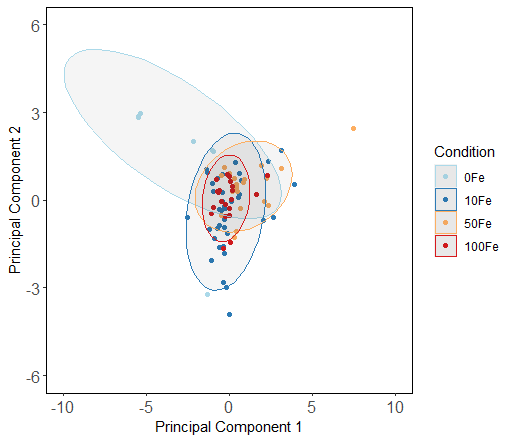


**Figure S2**. Principal component analysis (PCA) of cellular volume and content data from the tomography assay measured in *Symbiodium* sp. cells growing in different conditions.


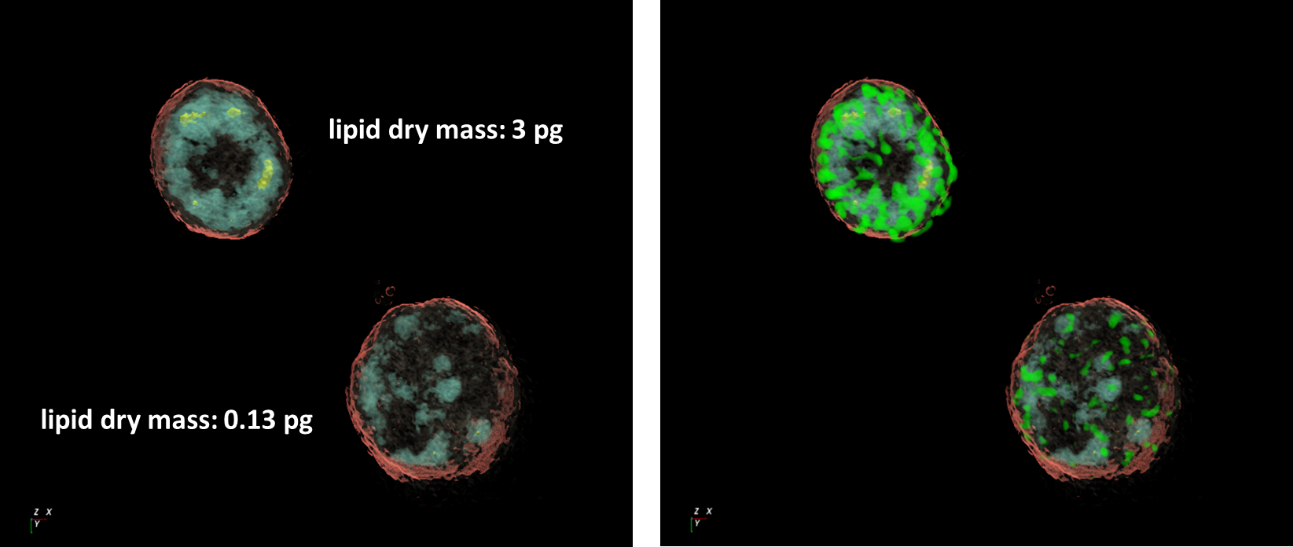


**b**

**a**

**Figure S3**: Digital holotomography images of *Symbiodinium* cells grown under 10 nM Fe concentration in the culture media. (a) 3D rendered image of the reconstructed RI distribution, at various viewing angles. The color coding is the same as in Figure 3; (b) Overlay of chlorophyll fluorescence image on the 3D RI rendering.
